# High-Resolution Mapping of Expression-QTLs Yields Insight into Human Gene Regulation

**DOI:** 10.1371/journal.pgen.1000214

**Published:** 2008-10-10

**Authors:** Jean-Baptiste Veyrieras, Sridhar Kudaravalli, Su Yeon Kim, Emmanouil T. Dermitzakis, Yoav Gilad, Matthew Stephens, Jonathan K. Pritchard

**Affiliations:** 1 Department of Human Genetics, The University of Chicago, Chicago, Illinois, United States of America; 2 Department of Statistics, The University of Chicago, Chicago, Illinois, United States of America; 3 Wellcome Trust Sanger Institute, Hinxton, Cambridge, United Kingdom; 4 Howard Hughes Medical Institute, Chevy Chase, Maryland, United States of America; The University of Queensland, Australia

## Abstract

Recent studies of the HapMap lymphoblastoid cell lines have identified large numbers of quantitative trait loci for gene expression (eQTLs). Reanalyzing these data using a novel Bayesian hierarchical model, we were able to create a surprisingly high-resolution map of the typical locations of sites that affect mRNA levels in *cis*. Strikingly, we found a strong enrichment of eQTLs in the 250 bp just upstream of the transcription end site (TES), in addition to an enrichment around the transcription start site (TSS). Most eQTLs lie either within genes or close to genes; for example, we estimate that only 5% of eQTLs lie more than 20 kb upstream of the TSS. After controlling for position effects, SNPs in exons are ∼2-fold more likely than SNPs in introns to be eQTLs. Our results suggest an important role for mRNA stability in determining steady-state mRNA levels, and highlight the potential of eQTL mapping as a high-resolution tool for studying the determinants of gene regulation.

## Author Summary

Individual phenotypes within natural populations generally exhibit a large diversity resulting from a complex interplay of genes and environmental factors. Since the advent of molecular markers in the 1980s, quantitative genetics has made a significant step toward unraveling the genetic bases of such complex traits, in particular by developing sophisticated tools to map the genomic locations of genes that affect complex traits. These regions are known as quantitative trait loci (QTLs). More recently, these tools have been extended to the study of gene expression phenotypes on a massive scale. In this paper, we used a previously published dataset consisting of expression measurements of 11,446 genes in human cell lines derived from 210 unrelated human individuals that have been genetically characterized by the International HapMap Project. Our article develops and applies a framework for determining the genetic factors that impact gene regulation. We show that these factors cluster strongly near to the gene start and gene end and are enriched within the transcribed region. Our approach suggests a general framework for studying the genetic factors that affect variation in gene expression.

## Introduction

Genetic variation that affects gene regulation plays an important role in the genetics of disease and adaptive evolution [1],[2],[3]. However, unlike protein-coding sequences, we still know little about how to identify the DNA sequence elements that control gene expression. It is still difficult to predict with any confidence which SNPs are likely to affect gene expression, without performing targeted experimental assays.

To address this gap, recent experimental and computational approaches have made progress on identifying elements that may be functional, for example through experimental methods that identify transcription factor binding sites [4],[5], by *in vivo* testing of possible enhancers [6] and by computational analysis of sequence data [7],[8],[9]. However, our understanding of the importance of different types of functional elements in gene regulation remains rudimentary.

As a complementary approach, genome-wide studies of gene expression are now starting to provide information on genetic variation that impacts gene expression levels [10]. Recent studies in a variety of organisms have shown that levels of gene expression are often highly heritable [11],[12],[13],[14], and that for many genes it is possible to map *cis*- and *trans*-acting factors using linkage [13],[15],[16],[17],[14] or association mapping [12],[18],[19],[20],[21]. Recent studies of experimental crosses in yeast and mice have used the locations of SNPs within eQTL genes to provide further information about the identity of functional elements [22],[23]. In studies of human lymphoblastoid cells, it has been reported that most strong signals of association lie within 100 kb of the transcribed region [12], and that eQTLs cluster roughly symmetrically around the TSS [20].

In this study, we applied a new Bayesian framework to identify and fine map human lymphoblast eQTLs on a genome-wide scale. In effect, we treat the SNP data as a tool for assaying the functional impact of individual nucleotide changes on gene regulation. Our analysis focuses on the impact of common SNPs on gene expression levels. By using naturally occurring variation, we test the effects of several million variable sites in a single data set. Our results provide a detailed characterization of the types of SNPs that affect gene expression in lymphoblast cell lines.

## Results

We analyzed gene expression measurements from lymphoblastoid cell lines representing 210 unrelated individuals studied by the International HapMap Project [24],[25]. These expression data, first reported by [19], were generated using the Illumina Sentrix Human-6 Expression BeadChip. For each sample we also used SNP genotype data from the Phase II HapMap Project, consisting of 3.3 million genotypes per individual [25].

After remapping the Illumina probes onto human mRNA sequences from RefSeq, we created a cleaned set of expression data for 12,227 distinct autosomal genes that had a unique RNA sequence in RefSeq (see Methods). For most analyses we removed 634 genes that had one or more HapMap SNPs within the expression probe and 147 very large genes (>500 kb), leaving us with a core data set of 11,446 genes.

We then set out to identify SNPs that affect measured mRNA levels in *cis*. As an operational definition, we considered the “*cis*-candidate region” to start 500 kb upstream of the transcription start site (TSS) and to end 500 kb downstream of the transcription end site (TES). Consistent with previous work [20],[12], our preliminary analysis found that most detectable eQTLs lie within this region.

Although the HapMap samples represent four different populations, originating from Africa, Europe and east Asia, our main analyses pooled the data into a single sample. To avoid false positives due to population-level expression differences [26],[20],[27], for each gene we transformed the African, European and east Asian expression data separately to standard normal distributions prior to combining the samples (Methods). Our rationale for combining samples was that we should achieve better power and better localization of signals than if we analyzed the populations separately. In doing so, we assume that functional variants usually have similar effects in different populations, an assumption that is parsimonious, and has empirical support [20], Figure S1. The overall results for analyses of individual populations are very similar (see Figures S2, S3, and S4).

### The Distribution of *cis*-Acting eQTLs

For each of the 11,446 genes, we tested for putative *cis*-acting eQTLs by regressing measured mRNA levels against SNP genotypes, independently for each SNP in the *cis*-candidate region, using a standard linear regression model. Consistent with previous reports [20], we found a substantial number of genes with strong evidence for containing at least one eQTL. A total of 744 genes (6.5%) had at least one SNP with a p-value <7×10^−6^. If the smallest p-value in each gene is treated as a summary statistic, this threshold yields a gene-level false discovery rate of 5% [28].

**Figure 1:**
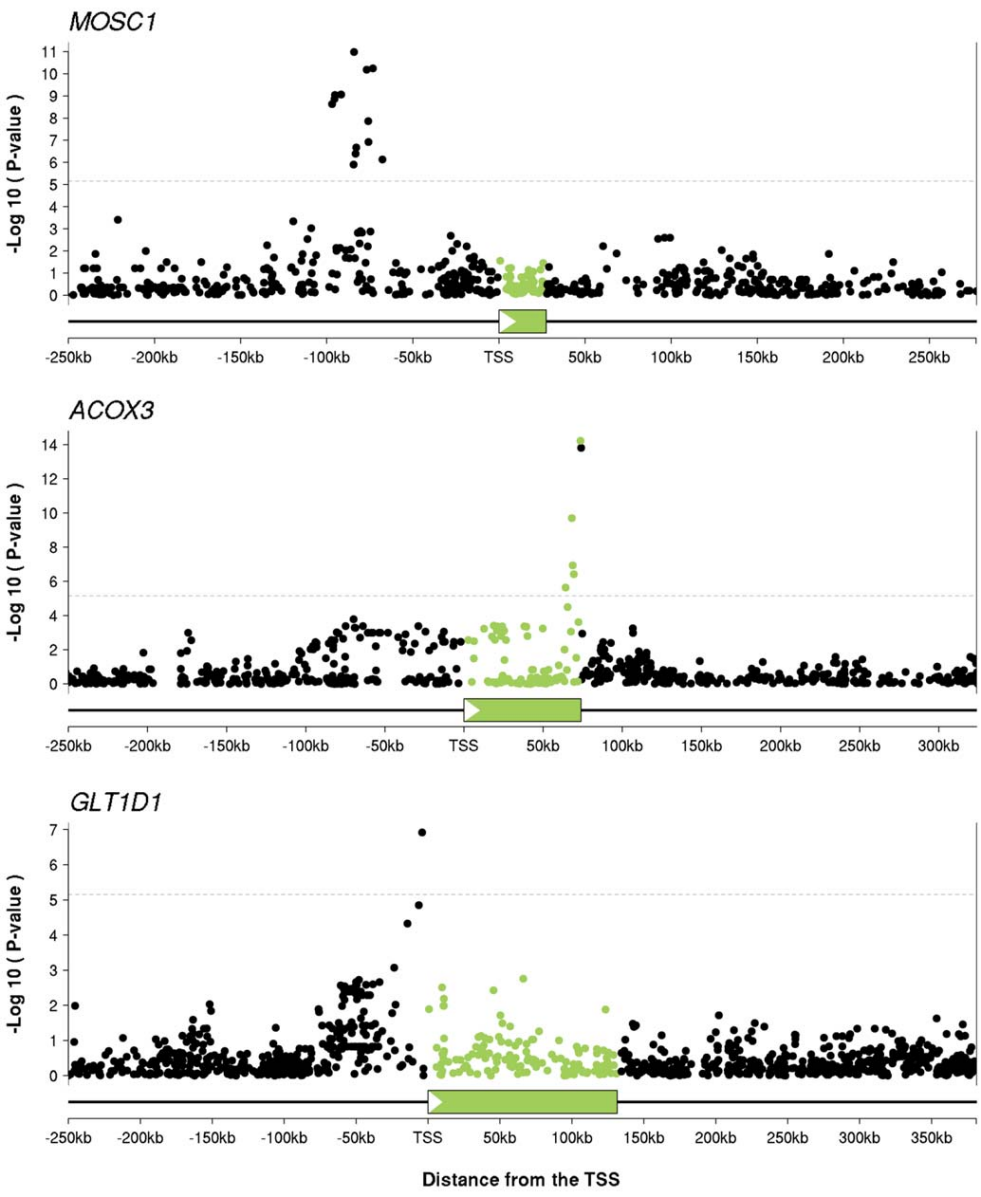
SNP association data often allow relatively precise localization of *cis*-eQTL signals. The plots show examples of eQTLs for three genes: *MOSC1*, *ACOX3* and *GLT1D1*. The x-axis on each plot indicates distance from the transcription start site. The transcribed regions are indicated by the green boxes and in all three plots the direction of transcription is left-to-right. For each SNP we plot the −log_10_(p-value) for association between genotype at that SNP and expression level of the gene. We use green to indicate SNPs that lie within the transcript of interest, and black for SNPs outside the transcript (this coloring is used for all the figures). The dotted line indicates the threshold for a gene-level FDR of 5% (p = 7×10^−6^).

We also observed that, in many cases, the SNPs most strongly associated with mRNA levels for a particular gene lie in a restricted region, allowing relatively precise localisation of eQTLs. Figure 1 plots examples of p-values in three genes, illustrating both the strong association signal that is often achieved, and the relatively localised nature of many of the signals (Figure S5).

Encouraged by the potential for these data to localise eQTLs, we next examined the distribution of the physical location of putative eQTLs within the *cis*-candidate region. For each gene with an eQTL (defined as having at least one p-value <7×10^−6^) we took the position of the *most* significant SNP as an estimate of the location of the functional site. In practice, we expect that the most significant SNP will sometimes be the actual functional site, but usually it will not since (1) HapMap contains only ≈1/3 of common SNPs [25]; (2) some eQTLs may be due to SNPs in LD with nearby copy number variants, though in practice few of the copy number variants known to be associated with expression are well-tagged by SNPs in these data (data not shown; [19]); (3) a non-functional SNP in strong LD with the functional site may have a smaller p-value by chance. Using simulations we estimate that the median distance between the functional SNP and the most significant SNP in our data is 7.5 kb (Figures S6 and S7). As expected, local recombination rates are strongly inversely correlated with the distance between the functional SNP and the most significant SNP (Figure S8).

**Figure 2:**
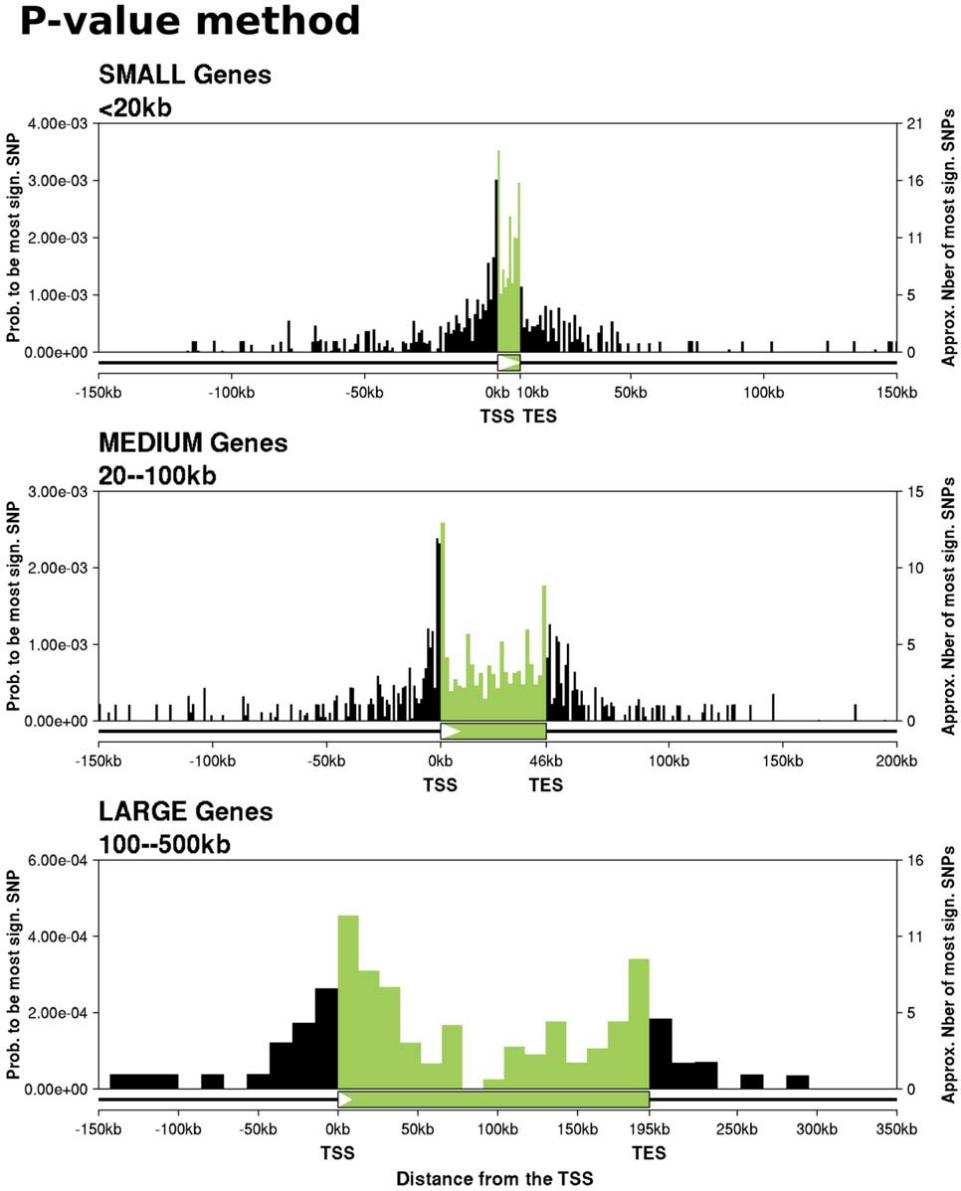
Locations of the most significant eQTL SNPs for small, medium, and large genes. Each plot shows, for genes with an eQTL, the distribution of locations of the most significant SNP. The x-axis of each plot divides a typical *cis*-candidate region into a series of bins as described. The y-axis plots the number of SNPs in each bin that are the most significant SNP for the corresponding gene and that have a p-value <7×10^−6^ divided by the total number of SNPs in that bin. The plotted data include an adjustment for the effect of unknown SNPs inside probes (Methods). SNPs outside genes are assigned to bins based on their physical distance from the TSS (for upstream SNPs), or TES (downstream SNPs). SNPs inside genes are assigned to bins based on their fractional location within the gene. There are 5372 “small” genes, of which 300 have an eQTL, 4489 medium genes (347 eQTLs), and 1585 large genes (94 eQTLs). The size of the schematic gene at the bottom of each plot indicates the average transcript length for that set of genes.

Figure 2 shows histograms of the locations of the most significant eQTL SNPs, as a function of gene size. (The plots incorporate a correction factor for the possibility of spurious signals due to undetected SNPs in the expression probes; see Methods.) Several interesting features emerge. First, the distribution of the most significant eQTL SNPs is roughly centered on the transcribed region. Second, nearly all such eQTL SNPs lie close to genes: we find relatively few that are >50 kb from the corresponding gene. Third, as shown in Figure S9, there is a significant enrichment of eQTL SNPs in exons compared to introns. We will return to this observation later in the paper.

Finally, for all three gene sizes, the highest density of eQTLs is around the TSS and immediately upstream of the TES, as reported previously in yeast [22]. The TSS peak was reported in a previous plot of these data [20], but in that previous analysis the TES signal peak was concealed due to the variability of gene lengths (see Figure S10). The TES signal is quite asymmetric: among genes with an eQTL, 10% (75) have the most significant eQTL in the 4 kb upstream of the TES, compared with just 4% (29) in the 4 kb immediately downstream.

### A Hierarchical Model of eQTLs in the *cis*-Candidate Region

While Figure 2 reveals the broad distribution of eQTLs and makes few modeling assumptions, it does not easily enable formal model testing about which aspects of gene structure (or other sequence features) are most important for generating eQTLs. Moreover, since the most significant SNP is not always close to the functional site, this approach can be expected to flatten out the true peaks of eQTLs and to increase the numbers of eQTLs that appear to lie far from the target genes.

Consequently, we next developed a Bayesian hierarchical modeling approach that solves many of these problems (see the Methods for further details). We considered a collection of models in which the parameters predict the prior probability that any given SNP in the *cis*-candidate region will be an “eQTN” (i.e., the functional *nucleotide* that creates an eQTL). Each model incorporates information about the physical locations of SNPs and, in some of our models, additional functional annotation of the SNPs. (Our calculations assume that the actual functional site is included in the HapMap genotype data; see below for further discussion.) The model parameters are estimated by maximizing the overall likelihood of the expression data, across all genes.

To implement our hierarchical approach, we switched to using Bayesian regression to test for association between SNPs and gene expression [29] (Methods). For each SNP in the *cis*-candidate region around a gene, we computed a Bayes factor that measures the relative support for the alternative hypothesis (the SNP is an eQTN) compared against the null (the SNP is independent of gene expression). For these data, the Bayes factors are highly correlated with p-values from standard linear regression. However, a key advantage of Bayes factors is that, combined with the prior probabilities specified by the model, they allow us to compute the posterior probability that each SNP is the actual eQTN.

The hierarchical model shares information across all genes about the distribution of signals and this in turn allows better weighting of which SNPs in individual genes are most likely to be eQTNs. For example, consider a hypothetical gene in which two SNPs that are associated with expression are in perfect LD (*r* ^2^ = 1). Suppose that one SNP is very close to the TSS, and the other is 30 kb upstream. In the p-value analysis, we would assign each of these SNPs 50% weight. In contrast, the hierarchical model downweights the upstream SNP because it is apparent from the overall data that eQTNs are much more abundant near the TSS, suggesting that the SNP near the TSS is much more likely to be responsible for the signal. Simulations show that the hierarchical model provides a more accurate profile of the distribution of eQTNs (see Figures S5 and S11).

Of course, some degree of complication is added by the fact that current HapMap data do not yet contain all SNPs. Therefore, the sites that we infer to be “eQTNs” in this study surely include many SNPs that are tags of nearby functional SNPs that are not in HapMap. This effect will systematically reduce our estimates of the importance of any particular factor in predicting eQTNs. In the case of factors relating to physical location (such as distance from the TSS) simulations show that this has a modest impact on spreading out the signal peaks that we observe, and that the overall distribution of signals is still estimated very well (see Figure S5, S11, and S12). In contrast, in the case of factors relating to functional categories (e.g., whether a SNP lies in a conserved element) we would expect the impact to be much more serious since functional elements are usually small and tag SNPs are unlikely to fall within the same element as a functional site. A second complication is caused by the possibility that undetected SNPs in the expression array probes might create spurious signals [30]. Our hierarchical model includes an explicit correction for this, using the 634 genes with a known SNP in the probe as training data.

### Distribution of eQTNs with Respect to the Transcribed Region

We first set out to get a more refined view of the distribution of eQTNs across the *cis*-candidate region. The basic versions of our hierarchical model described the positions of SNPs relative to a single “anchor” point such as the TSS. SNPs were grouped into discrete bins based on their distance upstream of the anchor, or downstream (treated separately). The bins nearest the anchor point were just 1 kb wide, to accommodate rapid changes in the rate of eQTNs, while more distant bins were wider (this improves the parameter estimates since the distant bins generally contain few eQTNs). Each bin was associated with a single parameter that relates to the proportion of SNPs in that bin that are eQTNs. The rationale for this model was that it would provide a good description of the data if, for example, the abundance of regulatory elements could be well predicted by distance from the TSS alone.

We also considered models with pairs of anchor points (e.g., the TSS and the TES). In those models, each SNP belonged to two bins, each corresponding to the distance from one anchor point. This model treats the probability that a SNP is an eQTN as the sum of an effect due to the first anchor plus an effect due to the second anchor. Recall that our gene set includes only genes with a single annotated transcript, so that this analysis does not incorporate alternative transcription start or end sites.

Table 1 compares eight different models using either a single anchor point (e.g., TSS or TES), or pairs of anchors (TSS and one other anchor). We used AIC (Akaike Information Criterion) to penalize the two-anchor models for the extra parameters that they use.

**Table 1:** Candidate models of eQTN locations, ranked by AIC.

Model	Log Likelihood Diff.	AIC Difference
**TSS+TES**	**0.0**	**0.0**
TSS+CDSE	−11.9	−11.9
TSS+Probe	−14.8	−14.8
TSS+TXMID	−58.5	−58.5
TSS+CDSMID	−63.5	−63.5
**TSS**	**−117.8**	**−66.8**
TSS+CDSS	−94.9	−94.9
TES	−330.7	−229.7

The table compares the performance of seven different hierarchical models of eQTN locations. In each model we used either a single “anchor” point to predict the locations of eQTNs (e.g., the location of the TSS) or two anchor points (e.g., the TSS and TES locations). The “TSS+CDSE” model uses the TSS and the coding sequence end locations as anchors; similarly “probe” refers to the location of the probe and “TXMID” is the midpoint of the transcript. The second and third columns compare the model listed on that line against the best model (TSS+TES), in terms of the difference in log likelihood (column 2) and the difference in Akaike Information Criterion (AIC, column 3). AIC penalizes the two-anchor models for 51 additional parameters compared to the one-anchor models.

In summary, the results provide compelling support for a model including both the TSS and TES over all other models (Table 1). Two other two-anchor models (namely TSS+probe location, and TSS+coding sequence end) also performed well, presumably because the Illumina probes and the coding sequence end positions are usually near to the TES. However, given that the TSS+TES model had by far the strongest support, we use this model in our subsequent analyses.

**Figure 3:**
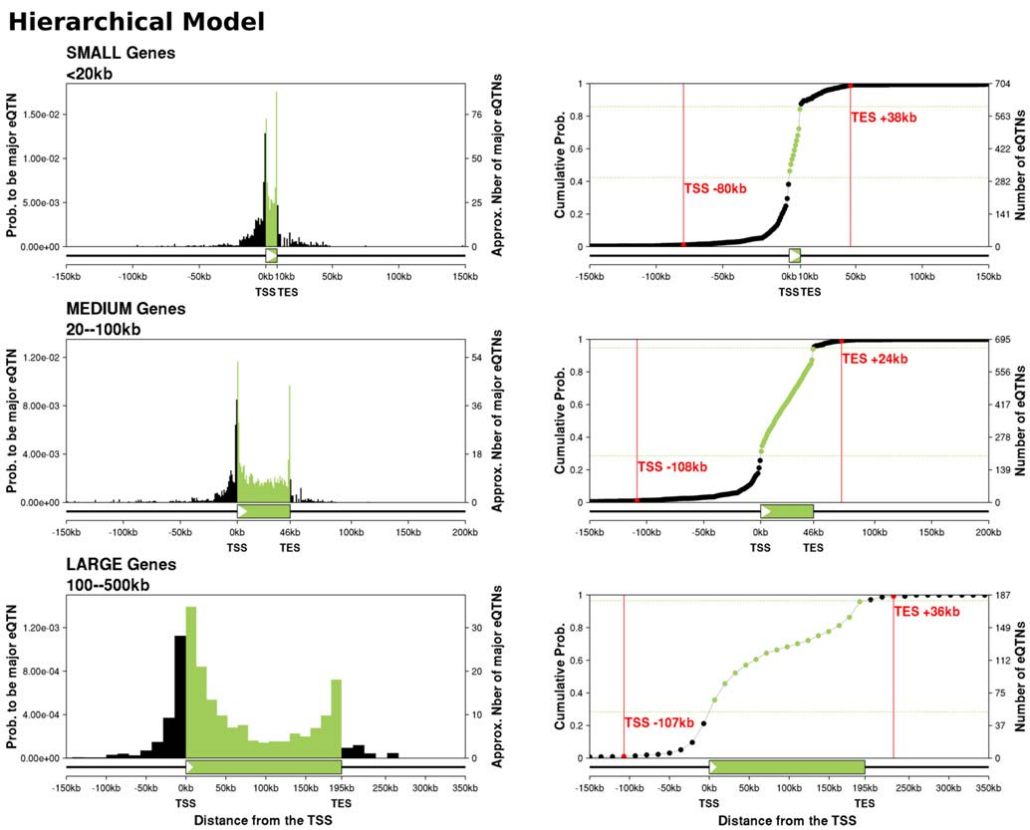
Locations of eQTNs, as estimated by the hierarchical model. The three left-hand panels plot the estimated fractions of SNPs in each bin that are eQTNs, using the posterior expected numbers of eQTNs in each bin from the hierarchical model. The right-hand panels plot the corresponding cumulative distributions of detected eQTNs, as a function of position across the *cis*-candidate region. The horizontal green lines indicate the gene boundaries; the vertical red lines indicate the 1% and 99% tails of the cumulative distributions. The numbers of eQTNs in each bin were calculated as the posterior expected numbers based on the SNP posterior probabilities from the hierarchical model.

We next replotted the locations of eQTNs, using the posterior probabilities estimated by the hierarchical model (Figure 3). Compared to the p-value-based analysis, the two strong peaks of signal near the TSS and TES are considerably strengthened. Also, in the hierarchical model, the level of background signal upstream and downstream of the gene is greatly reduced, presumably because most of the background signal in the p-value analysis can be explained as resulting from LD with SNPs near the TSS and TES. The hierarchical model estimates that the total number of eQTLs is considerably larger than the number that we detected by linear regression at the rather stringent false discovery rate of 5% (1586 vs. 744). This difference is partly because the hierarchical model adds fractional probabilities for eQTLs that have only partial support for being true eQTLs, and partly because the hierarchical model is more sensitive to signals in locations that are likely *a priori*.

Another view of the hierarchical model results is shown in the cumulative plots in Figure 3, which plot the cumulative distribution of eQTNs across the gene region. Most eQTNs lie close to the gene, with less than 7% of the detected *cis*-eQTNs located more than 20 kb outside the gene. Overall, there are about 3-fold more eQTNs in the upstream region of the gene (5′ of the TSS) than downstream (3′ of the TES) (30% vs. 9%).

**Figure 4:**
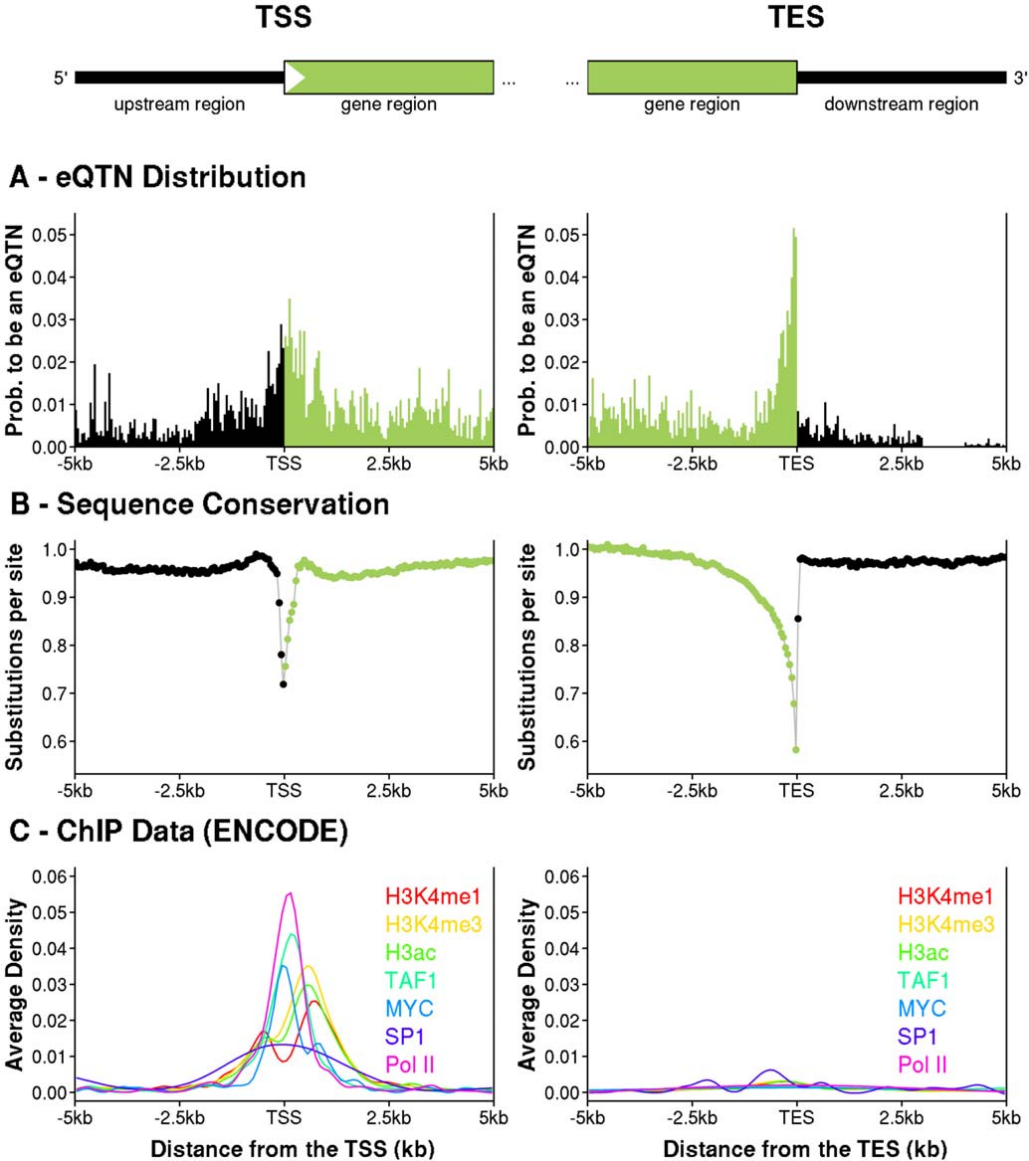
Fine-scale structure of eQTN peaks near the TSS and TES, and comparison to average sequence conservation and transcription factor binding density. The left- and right-hand columns show data for 5 kb on either side of the TSS and TES, respectively (averaging across all gene sizes). Locations inside genes are colored green and outside genes are black. A. Posterior expected fractions of SNPs in each bin that are eQTNs, as estimated by the hierarchical model (see Methods). Each bin is 50 bp wide. B. The average number of substitutions per base pair across the phylogeny of seven mammalian species for all 11,446 genes analyzed in this study (see Methods). Coding sequences were excluded. Each data point is the average across a 50 bp bin. C. The average density of factor binding fragments for seven factors related to transcription initiation and studied by ENCODE using ChIP-chip in 1% of the genome [4]. The TSS part of panel C replots data (H3K4me1, H3K4me3, H3ac, MYC and Pol II) from Figure 5 of [4].

We next investigated the peaks of signal near the TSS and TES in more detail, using a finer bin partition close to the TSS and TES (see Figure 4A and Methods). At this finer scale, the TES signal is extremely sharply peaked over a region of just ∼100 bp immediately upstream of the TES. The data strongly reject a model in which the signal is symmetric around the TES (p = 3×10^−7^). In contrast, the TSS signal is more spread out, and spans both sides of the TSS. There is no evidence of asymmetry in the TSS signal (p = 0.34).

We also observed that the TSS and TES peaks both correspond with two parts of the typical gene structure that, averaging across all 11,446 genes, tend to be highly conserved across the mammalian phylogeny (Figure 4B). The correspondence of the two eQTN peaks with the peaks of conservation suggests that there may be a causal link between these two types of signals. We propose that the sequence conservation reflects, at least in part, the roles of these two locations in regulating mRNA levels, though further work will be needed to verify the connection.

Similarly, the TSS peak also matches up closely with the peak binding densities of a collection of transcription factors that are involved in transcription initiation (reported previously by the ENCODE group, based on ChIP-chip data collected for a set of regions spanning ∼1% of the genome [4]). As might be expected, the ENCODE data identified almost no transcription factor binding near the TES. We return to these latter observations in the Discussion.

### Distribution of eQTNs with Respect to Functional Annotation

We next used our hierarchical model to examine the impact of various types of functional annotation on the probability that a SNP is an eQTN. We first classified SNPs that lie inside genes into categories based on the exon/intron structure (e.g, first, coding and last exons; first, internal, and last introns; Figure S13). In order to make the model fully identifiable, we estimate the effect of each annotation *relative* to the abundance of eQTNs in internal introns (as this category has the greatest number of SNPs). Since gene position is highly predictive of eQTN abundance, we controlled for SNP position using the TSS+TES model. In effect, the hierarchical model now tests whether the annotation adds any predictive value beyond the basic position information. As noted above, incomplete SNP ascertainment in HapMap means that we will generally underestimate–perhaps substantially–the impact of relevant annotations.

**Figure 5:**
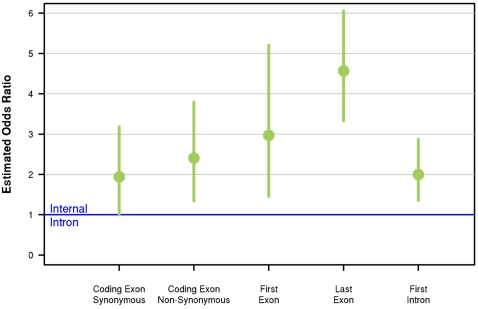
Expression-QTNs are under-represented in coding sequence introns, even after controlling for position effects. The plot shows the odds ratios for the probability that a SNP in a particular part of the gene (e.g., coding exon) is inferred to be an eQTN, relative to that probability for a SNP in an “internal” intron (i.e., an intron within the coding sequence). The odds ratios are estimated using the hierarchical model with internal introns fixed at a value of 1, and control for SNP position using the TSS+TES model. The vertical bars show 95% confidence intervals.

The main result of this first analysis is that internal introns have a deficit of eQTNs, compared to coding exons, as well as first and last exons and introns (Figure 5, Table S1). For example, SNPs in coding exons are ∼2-fold more likely than SNPs in internal introns to be eQTNs. First introns are also relatively enriched for eQTNs compared to internal introns (controlling for position). However, since the total amount of sequence contained in introns vastly exceeds that in exons, 53% of genic eQTNs lie in internal introns compared to 10% in coding exons (see Table S1). SNP density differs slightly between exons and introns, but not nearly enough to generate a 2-fold difference in eQTN abundance (Table S2). Overall, the hierarchical model that includes the gene structure annotation as well as position effects relative to the TSS and TES is substantially better than the TSS+TES-only model (by 7 units of AIC).

We then considered the impact of a variety of other types of SNP annotation (see Methods and Figure S14). None of these annotations shows convincing signals of enrichment (Table S3). We estimate a 1.9-fold enrichment of eQTNs inside conserved noncoding elements, as might be expected if these identify functional elements, however the 95% confidence interval narrowly overlaps 1. We also tested for an enrichment of eQTNs at computationally predicted microRNA binding sites, reasoning that SNPs in these binding sites might affect mRNA degradation. We found a suggestive, but non-significant, enrichment of eQTNs in these sites (1.4-fold). It is unclear whether the absence of significant effects in these analyses indicates that these types of annotation are not strongly associated with eQTNs or instead reflects the incompleteness of HapMap and the limitations of current functional annotations.

Finally, based on ENCODE results showing that the promoter regions of genes with CpG islands tend to have more accessible chromatin and greater occupancy by transcription factors [4], we predicted that CpG status might also provide relevant annotation. Indeed, we find that genes with a CpG island spanning the TSS are expressed at higher average levels, and are ∼50% more likely than genes without a CpG island on the TSS to have a *cis*-eQTN (15% vs 11%). This effect is consistent with the observation that genes with CpG islands are more likely to be expressed in a wide range of tissues than are genes without CpG islands [31]. After adjusting for the different overall rates of eQTNs, the distribution of signal locations in the two classes of genes is very similar (Figure S15).

## Discussion

Cells use a variety of mechanisms at the transcriptional and translational levels to regulate gene expression. Transcription initiation is controlled by the interaction between transcription factors and cofactors with a set of *cis*-acting regulatory elements including core and proximal promoters that lie close to the TSS, as well as enhancers, silencers and boundary elements that may act at a distance [32],[33],[34],[35]. Initiation is also affected by epigenetic properties of the DNA such as chromatin condensation and DNA methylation. After transcription initiation, mRNA levels can also be regulated during mRNA elongation or splicing and by mRNA stability and degradation. However, for most genes, transcription initiation is usually thought to be the principal determinant of the overall mRNA gene expression profile [34],[35].

Consistent with the importance of transcription initiation, we found a strong peak of eQTNs near the TSS, with 33% of eQTNs lying within 10 kb of the TSS. Many of these eQTNs are likely to be polymorphisms that change the binding strength of transcription factor binding sites, thereby affecting the rate of transcription [22]. We also found that eQTNs are distributed roughly symmetrically around the TSS, with the peak density in ∼1 kb on either side (c.f. [20]). Our results at the TSS are consistent with recent observations by the ENCODE team that the peak density of transcription factor binding is centered on the TSS (Figure 4C). These observations indicate that empirical scans for regulatory variants that only look upstream of the core promoter [e.g., 13],[36] may often miss important sites of regulation.

In addition to the peak of eQTN signals near the TSS, we were intrigued to find a second, similarly strong peak near the TES, as seen previously in yeast [22]. This peak is more concentrated than the TSS peak, localizing immediately before the TES, and dropping extremely rapidly after the TES. We also found that, after controlling for position effects, SNPs in exons are consistently more likely than SNPs in internal introns to be eQTNs. These observations suggest that an important fraction of eQTNs may affect properties of the transcript, rather than of the DNA sequence. We hypothesize that these eQTNs are typically polymorphisms that affect transcript stability or the rate of transcript degradation [37],[38],[39],[40],[41]. In contrast to transcription initiation, mRNA stability has been less widely studied and we still have an incomplete picture of the mechanisms that determine transcript persistence. One such mechanism is the hybridization of microRNAs to single strand transcripts, thereby exposing them as targets for degradation. Hence a SNP that creates or disrupts a match between a microRNA and the transcript might affect the rate of degradation [40]; however we did not find a significant enrichment of eQTNs in predicted microRNA binding sites.

An alternative explanation for the overrepresentation of eQTNs in exons is that in some cases these may cause alternative splicing of the exon containing the expression probe, thereby changing measured expression levels. In particular, SNPs in the last exon might sometimes affect the location of the TES [21], perhaps even deleting the expression probe from the transcript. While this mechanism probably accounts for some of the data, we do not believe it is the main explanation for several reasons. First, we found that the TSS+TES model was significantly better than the TSS+probe model. If the effect was mainly due to SNPs that affect alternative splicing of the exon containing the probe, we anticipate that those SNPs would usually lie nearer to the probe than to the TES. In that case the TSS+probe model should have performed best. Second, in a separate analysis, we observed an enrichment of signals near the TES in Affymetrix exon array data when we combined data across probes from multiple exons (results not shown, data from [21]). Third, the striking peak of sequence conservation near the TES (Figure 4B) indicates that this is a region with strong functional significance, presumably due to an important role in gene regulation.

Our results also imply that surprisingly few eQTNs with large effects lie far upstream of the TSS (or downstream of the TES): for example, just 5% of the eQTNs that we detected were more than 20 kb upstream of the TSS. These results are consistent with data showing that most transcription factor binding sites are near the TSS [4]. However, since our method focuses on the major eQTN in each gene, we may under-estimate the abundance of distant eQTNs if these typically have smaller effect sizes ([12]). By focusing on SNPs, our analysis may miss the impact of other types of variation–such as copy number variation–that might plausibly exert effects over different physical scales. It is also possible that more distant elements are less likely to be disrupted by single nucleotide changes. Finally, it will be important to revisit the questions that we considered here in a range of other tissues. By studying cell lines, we may underestimate the importance of long-range enhancers that turn genes on or off depending on conditions outside the cell (e.g., during development).

In summary, our results show that eQTL studies provide a remarkably high-resolution tool for identifying variants that affect gene expression. A major strength of the eQTL approach is that, unlike other experimental techniques that are more targeted, the eQTL approach is agnostic about the mechanism of action of the functional variants, provided only that they are encoded in the DNA sequence (as opposed to epigenetic factors, for example). Hence, eQTL studies can provide a relatively unbiased view of the importance of different types of regulatory mechanisms. Moreover, as the cost of genome sequencing drops, it will soon be possible to conduct these analyses with nearly complete ascertainment of variation, potentially providing this approach with the resolution to study the sequence level determinants of gene expression. We anticipate that eQTL mapping will make an essential contribution to our understanding of human gene regulation.

## Methods

### Genotype Data

We analyzed genotype and expression data from 210 unrelated individuals studied by the International HapMap project [24],[25]. These include 60 Yoruba (YRI) and 60 CEPH (CEU) parents, and 45 unrelated Chinese (CHB) and 45 unrelated Japanese (JPT) individuals. We used the HapMap Phase II genotype data, release #21 (phased and with missing data imputed). We used data from the 22 autosomal chromosomes only, giving a total of 3,304,587 SNPs. Since allele frequencies in CHB and JPT are extremely similar [24], these two samples were treated as a single analysis panel of 90 Asians (“ASN”).

### Gene Expression Data

We used gene expression levels that were measured previously in lymphoblastoid cell lines from all 210 unrelated individuals, using Illumina's human whole-genome expression array (WG-6 version 1) [19]. We downloaded the data that were normalized first by quantile normalization within replicates and then median normalized across all HapMap individuals [19] [ ftp://ftp.sanger.ac.uk/pub/genevar/].

Since mean expression levels at many loci differ between the HapMap populations [26],[42],[20],[27], there is a potential for spurious eQTLs in the combined sample due to population structure. To control for this effect, we applied a normal quantile transformation to the data for each gene, within each HapMap population (ASN, CEU, YRI), prior to combining the samples. That is, for each gene, separately in each population, we transformed the *r*th largest gene expression value to the (*r*−0.5)/*n*th quantile of the standard normal distribution, where *n* is the number of individuals with gene expression data from that population [29]. By forcing each population to have the same distribution of expression values, we avoid concerns about spurious associations due to allele frequency differences between the HapMap populations. (Note that the overall results within populations are very similar; Figures S2, S3, and S4.) This normalization also reduces the effect of outlying expression values on the regression [29].

### Selection of Genes and Probes

We used BLAT [43] to map the 47,294 Illumina array probes onto human RNA sequences from RefSeq (hg18) [44]. The accession numbers of the RNA sequences were mapped against the Entrez Gene database and all probes that mapped with greater than 90% identity to multiple genes were discarded. Of the remaining probes we retained only those with exact matches to a unique gene, leaving us with 19,536 valid probes. Of these, we kept the 13,244 probes for which the gene has a single RNA accession in the RefSeq database. This was done to simplify the analysis by avoiding genes with multiple splice forms or multiple annotated start sites, etc. These 13,244 probes map to 12,227 unique autosomal genes.

Of these 12,277 genes, 85% contained exactly one probe. For the genes with multiple probes, we analyzed only a single probe, selecting the probe nearest to the 5′ end of the gene. We selected this probe because overall the probes are strongly biased towards the 3′ end of the gene, and we wanted to reduce this bias as far as possible. Then, we removed 634 genes for which there was at least one HapMap SNP inside the probe since it is known that such SNPs can impact the measured expression level [30]. Finally, 147 very large genes (size greater than 500 kb) were discarded, leaving our core data set of 11,446 genes.

### Gene Structure and SNP Annotation

Gene structure annotation was obtained from the RefSeq gene table [44] for human genome build 35 (hg17). For each gene the TSS and the TES genomic locations were obtained from the fields “Transcription start position” and “Transcription end position” of the RefSeq table, respectively. We checked the genomic positions of the TSSs against dbTSS, a database of experimentally-determined TSSs, [45] and found no differences among the 84% of gene transcripts in our data set that are also in dbTSS. We defined the CDS (coding sequence) to be everything between the translation start and stop positions defined by the fields “cdsStart” and “cdsEnd”, respectively, of the RefSeq Table. We then assigned every genic SNP to one of 8 mutually exclusive gene-related annotations (see Figure S16):

- First (non-coding) exon. If the gene has at least 2 exons, this is the part of the first exon that is not located inside the CDS. If the gene has only one exon, we do not consider it to have a *first* exon.
- First intron. If the gene has at least 2 exons, this the intron following the first exon, provided that it is not located inside the CDS. Otherwise there is no first intron.
- Noncoding exon. This is any part of an exon located outside the CDS region and excluding the first and last exons.
- External intron. This is an intron located outside the CDS region and excluding the first and the last introns.
- Coding exon. This is any part of an exon located inside the CDS region. Note that exons containing the translation start or stop generally contain both coding exon and noncoding (or first/last) exon. Coding SNPs were further subdivided into synonymous and nonsynonymous, according to their annotation in dbSNP.
- Internal intron. This is an intron located inside the CDS region.
- Last intron. If the gene has at least 2 exons, this is the intron preceding the last exon, provided that it is not located inside the CDS. Otherwise there is no last intron.
- Last (noncoding) exon. If the gene has at least 2 exons, this is the part of the last exon that is not located inside the CDS. Otherwise there is no last exon.

We also included annotations that indicate whether a SNP is in the following special categories: SNP is in a (1) CpG island; (2) conserved noncoding region; (3) predicted *cis*-regulatory module; (4) predicted micro-RNA binding site; or that (5) a predicted binding site of the CTCF insulator protein lies between the SNP and the TSS. See the Supplementary Methods (Text S1) for further details.

Finally, note that in our analysis design, each SNP is tested for association with every gene that is within 500 kb. This means that typical SNPs contribute data to multiple genes. Our analysis treats these multiple tests as independent, which is likely a good approximation since we identified only five SNPs that are eQTLs for > one gene in *cis*.

### Statistical Analysis

#### Notation

The data consist of SNP genotypes and gene expression measurements for *n* individuals at each of *K* genes. Let *y_ik_* denote the normalized gene expression data for individual *i* (*i* in 1,…, *n*) at gene *k* (*k* in 1,…, *K*). *Y_k_* will denote the vector of gene expression values (*y_.k_*) across the *n* individuals at gene *k*.

Next, let *M_k_* be the number of genotyped SNPs in the *cis*-candidate region of gene *k*. We denote the entire matrix of genotype data for these *M_k_* SNPs with the vector *G_k_*, and individual genotypes as *g_ijk_* for individual *i* at SNP *j* of gene *k*. Genotypes are coded as having 0, 1, or 2 copies of the minor allele.

#### P-Value Method

In the first part of the paper we used standard linear regression to test the gene expression data at each gene for association with SNPs in the *cis*-candidate region, as follows. The effect of individual *i*'s genotype at SNP *j* (*g_ijk_*) on his/her gene expression level (*y_ik_*) is assumed to follow an additive linear model:
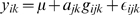
(1)where *μ* is the mean expression level at that gene for individuals with *g* = 0, where *a_jk_* is the additive effect of the minor allele at SNP *j* and *ε_ijk_* is the residual. A standard p-value from a 1 df test can then be obtained for the hypothesis that SNP *j* is an eQTN for gene *k* (*a_jk_* ≠ 0).

We used the following procedure to generate the results plotted in Figure 2. For each gene with expression data we assigned each SNP in the *cis*-candidate region to a single bin (see below). Let *m* be the total number of SNPs that fall into bin *b*, summing across all genes. (Note that most SNPs are in the *cis*-candidate regions of multiple genes and hence can contribute data to multiple bins.) Next, for each gene, we tested every SNP for association with gene expression. If the p-value of the most significant SNP was <7×10^−6^ then we considered this to be one “signal” in the bin that this SNP lies in. (Note that the results are robust to the choice of the p-value cutoff; Figures S17 and S18.) For genes in which the smallest p-value was shared by *n*>1 SNPs, we considered that the signal was divided equally among the *n* most significant SNPs (i.e., a fraction 1/*n* of the signal was assigned to each SNP). Suppose that, by this way of counting, there are *s* signals in bin *b*.

Prior to reporting the data, we also applied a correction for the possibility of spurious signals due to ungenotyped SNPs in the expression array probe. We used the 634 genes with a known HapMap SNP inside the probe to create a profile of the abundance of spurious signals as a function of distance from the probe. This profile was used to adjust the observed number of signals, *s*, to a corrected number *s*′, that removes the predicted number of spurious signals in each bin (see Figure S19 and Text S1 for details). In practice, we estimate that the contribution of spurious signals does not substantially change the overall uncorrected distribution of signals. Finally, we computed the fraction of most significant SNPs in bin *b* as *s*′/*m*.

#### Bin Definitions

To display the distribution of signals in Figures 2 and the left panel of Figure 3 we subdivided the *cis*-candidate region into discrete bins as follows. First, since there is dramatic variation in gene sizes, we analyzed genes in three separate categories based on transcript length: small genes (0–20 kb), medium genes (20–100 kb) and large genes (100–500 kb). Then, within each gene size category we divided the entire *cis*-candidate region into a series of bins, anchored at the TSS and TES. SNPs outside the transcript were assigned to bins based on their distance from the TSS (for the upstream region) or TES (downstream). Bins outside the transcript were 1 kb wide for small and medium genes and 15 kb wide for large genes. Transcribed regions were split into fixed numbers of bins: each small gene was split into ten bins of equal size, medium genes into 25 bins and large genes into 15 bins. Hence, bins inside the transcript indicate the fractional location of SNPs relative to the TSS and TES, and the physical sizes of the bins vary across genes. The bin sizes were chosen so that the average physical sizes of internal and external bins are roughly the same within each gene size category.

### Hierarchical Model

We present here an overview of the hierarchical model. Complete details on the models are provided in the Supplementary Methods section (Text S1).

#### Bayesian Regression Model

The hierarchical model applies the Bayesian regression framework of Servin and Stephens [29]. The effect of individual *i*'s genotype at SNP *j* (*g_ijk_*) on his/her gene expression level (*y_ik_*) is assumed to follow a linear model:
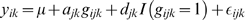
(2)where *μ* is the mean expression level at that gene for individuals with *g* = 0, and where *a_jk_* and *d_jk_* are the additive and dominance effects of the minor allele at SNP *j*. The residual, *ε_ijk_*, is assumed to be N_(0,1/*τ*)_ and independent for each *y_ik_*, where 1/*τ* is the variance of expression levels within each genotype class. The indicator function *I*(*g_ijk_* = 1) is defined as 1 if the genotype is heterozygous (*g_ijk_* = 1) and 0 otherwise.

Let 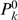 denote the probability of the expression data *Y_k_* under the null hypothesis that there are no *cis*-eQTNs in gene *k* (i.e., *a_jk_* = *d_jk_* = 0 for all *j*). Similarly, let 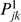 denote the probability of the expression data *Y_k_* assuming that SNP *j* is the eQTN. In this case, the effect sizes *a_jk_* and *d_jk_* are modeled as being drawn from mixtures of normal distributions centered on 0 (see Text S1 for details). The Bayes factor (BF) for SNP *j* in gene *k* is defined as
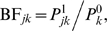
(3)and measures the relative support for the hypothesis that SNP *j* is an eQTN for gene *k*, versus the null hypothesis. We use priors on effect sizes that allow the BF to be calculated analytically (see Text S1).

#### The Hierarchical Model

We describe first the basic version of our hierarchical model. All the results presented in this paper additionally include a correction for the possibility that genes might show signals due to undetected SNPs in the probe. We describe that extension later in the Methods, briefly, and in detail in the Supplementary Methods (Text S1).

Our basic model assumes that there are two mutually exclusive categories of genes. With probability Π_0_ there is no eQTN in the *cis*-candidate region, and with probability Π_1_ = 1−Π_0_ there is a single eQTN. Then the likelihood of the expression data at gene *k* is
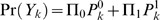
(4)where 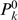 denotes the probability of the expression data *Y_k_* given that there is no eQTN in gene *k* and 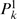 denotes the probability of the expression data given that there is exactly one eQTN. Note that our model allows for at most one eQTN per gene. If in fact there is more than eQTN, our model will usually assign the signal to the strongest of these. In practice, we see little variation in average effect size as a function of location, so this modeling simplification is unlikely to seriously distort the results.

Given that there is a single eQTN in gene *k*, the probability of the observed expression data, 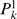, can be written as
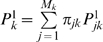
(5)where 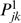 is the probability of the expression data given that SNP *j* is an eQTN, and π*_jk_* is the prior probability that SNP *j* is an eQTN, given that exactly one SNP in gene *k* is an eQTN.

A key feature of the hierarchical model is that the probability that SNP *j* is an eQTN, π*_jk_*, is allowed to depend on the physical location of SNP *j* relative to one or more “anchor” points, and other relevant annotations (see Text S1). Suppose that we consider *L* different kinds of annotation, and let the indicator *δ_jkl_* equal 1 if SNP *j* at gene *k* has the *l*th annotation, and equal 0 otherwise. Then define
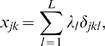
(6)where Λ = (λ_1_,…,λ*_L_*) is a vector of annotation effect parameters. We use a logistic model to relate π*_jk_* to these annotation indicators, namely,
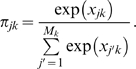
(7)


As detailed in the Supplementary Methods (Text S1), we parameterized the effect of distance from the anchor locations using a series of discrete bins that represent absolute physical distance from the relevant anchor. The bins nearest to the anchor are 1 kb wide, and increase in width to 10 kb and finally 100 kb with increasing distance from the anchor. For the two-anchor models, each SNP belongs to two position bins, each of which indicates distance from one anchor.

#### Likelihood for the Hierarchical Model

Substituting the above expressions for 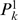 into (4), the likelihood for the hierarchical model is
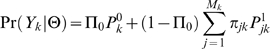
(8)

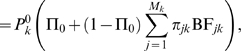
(9)where Θ denotes the model parameters and BF*_jk_* is the BF from the Bayesian regression (3). To be explicit, the model parameters Θ include the annotation parameters Λ, the proportion Π_0_ and other parameters related to the Bayes factor computation (see Text S1). The likelihood of the entire data set is the product of (9) across all *K* genes. We fit the hierarchical model by maximizing the log-likelihood
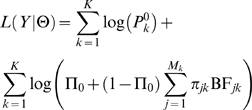
(10)with respect to the model parameters Θ. (Note that the first term, involving 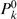 does not depend on Θ, and so need not be evaluated.)

#### Accounting for the Effects of SNPs in Probes

Since undetected SNPs in the probe sequence sometimes generate eQTLs, the results that we report include a modification to account for this effect. We used the 634 genes that have a known SNP in the probe region as training data to help parameterize the model. We assume that these represent ∼1/3 of all probes with common SNPs [25].

Suppose that with probability 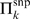 there is a gene inside the probe sequence (this is set to 1 for the training data), and suppose that when there *is* a SNP in the probe, there is a probability Π*_s_* that this generates a spurious signal. Then let 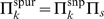 be the probability of a spurious signal. We consider that we are only interested in real signals if there is no spurious signal, so we write the probability of the data as
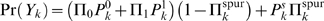
(11)where the first term is the likelihood when there is no spurious signal (as in Equation 4), and where the second term gives the likelihood (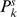) when there is a spurious signal.

#### Likelihood Maximization

To maximize [10] we used an iterative strategy based on a point-by-point golden maximization strategy [46]. To speed convergence of the maximization process, we initialized the parameters using naive estimates of the λs based on the logarithm of the odds ratio computed assuming Π_0_ = 0.

#### Posterior Probabilities

Once the likelihood has been maximized, we can compute the posterior probability of a given SNP *j* to be an eQTN for gene *k*. In the case without spurious signals this is
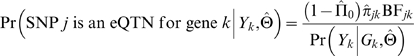
(12)and the general version is given in the Supplementary Methods (Text S1).

### Sequence Conservation and Transcription Factor Binding

To compute the average sequence conservation as a function of position for Figure 4B, we estimated the average number of substitutions per site across the phylogeny of seven mammalian species (human, chimpanzee, macaque, mouse, rat, dog, and cow), using data and alignments from the UCSC browser. This was done for the main set of 11,446 genes analyzed in this paper. For each gene, 5 kb on each side of the TSS (and separately for the TES) was split into non-overlapping 50-bp bins. We then concatenated all the sites across all genes that lay in the same bin. After excluding sites in coding exons we estimated the average number of substitutions at each site using *baseml*, a program in the PAML package [47].

We obtained results on transcription factor binding density using ChIP-chip data collected by the ENCODE project (4). We used data for eight transcription factors that showed large numbers of binding fragments at a 1% false discovery rate in the ENCODE study. The left-hand panel of Figure 4C is essentially a replotting of data presented in Figure 5 of (4), while the right-hand panel shows analogous data plotted with respect to the TES.

#### Software Availability

The methods reported here are implemented in the package *eQTNMiner*, which is available from JBV on request.

## Supporting Information

Figure S1 About 60% of the eQTNs are shared between at least two populations. Venn diagram of the set of eQTNs detected separately in each population. To generate the diagram, we admitted a SNP to the analysis (as an eQTL) if either the p-value in the combined sample (pooling the 3 populations) is lower than 7×10^−6^ or the p-value in a single population is lower than the p-value cutoff corresponding to a gene FDR of 5% within each population. We then considered two populations to share an eQTL if any single population has a p-value <1×10^−2^. Finally, for each gene having at least one such eQTL, we defined the eQTN as the SNP with the largest number of shared populations (sharing weight between multiple SNPs if there is a tie).
(0.12 MB PNG)

Figure S2 Expression QTNs in the combined Japanese plus Chinese analysis panel (ASN) show similar patterns to those in the full data. The left panel (p-value method) was prepared in the same way as Figure 2 of the main paper and the right panel (hierarchical model with TSS+TES) was prepared in the same way as Figure 3 (left panel) of the main paper. Both display results analyzing only the Asian data. For the left panels we used a p-value cutoff of 1.25×10^−5^ obtained by permutations when analyzing only the Asian data and corresponding to a gene FDR of 5%.
(0.43 MB PNG)

Figure S3 Expression QTNs in the European-derived sample (CEU) show similar patterns to those in the full data. The left panel (p-value method) was prepared in the same way as Figure 2 of the main paper and the right panel (hierarchical model with TSS+TES) was prepared in the same way as Figure 3 (left panel) of the main paper. Both display results analyzing only the European data. For the left panels we used a p-value cutoff of 3.5×10^−6^ obtained by permutations when analyzing only the European data and corresponding to a gene FDR of 5%.
(0.46 MB PNG)

Figure S4 Expression QTNs in the Nigerian sample (YRI) show similar patterns to those in the full data. The left panel (p-value method) was prepared in the same way as Figure 2 of the main paper and the right panel (hierarchical model with TSS+TES) was prepared in the same way as Figure 3 (left panel) of the main paper. Both display results analyzing only the Nigerian data. For the left panels we used a p-value cutoff of 3.825×10^−6^ obtained by permutations when analyzing only the Nigerian data and corresponding to a gene FDR of 5%.
(0.43 MB PNG)

Figure S5 Illustration of the ability of the HM to accurately estimate the distribution of eQTNs when all the actual eQTNs are genotyped. This figure is based on a simulated dataset assuming that for all genes the actual eQTN is genotyped (see Text S1). In both panels the black histograms represent the number of actual eQTNs using 1 kb bins anchored from the TSS (this is identical for both panels). A. P-value method: the green curve displays the number of most significant SNPs detected by the p-value method. As expected, due to LD and the stringency of the p-value cut-off, the profile is less peaked than the actual distribution. B. Hierarchical model: using our hierarchical model with the TSS-only model (see Methods) we are able to catch most of the actual eQTNs. The red curve indicates the expected number of eQTNs computed using the posterior probabilities from the hierarchical model. Notice that the hierarchical model provides a better picture of the distribution of signals.
(0.15 MB PNG)

Figure S6 50% of the most significant SNPs lie within 7.5 kb of the actual eQTNs. Both panels are based on the results from the p-value method applied to a simulated dataset (see Text S1). The top panel plots the histogram of the fraction of most significant SNPs as a function of distance from the actual eQTNs. The bottom panel plots the corresponding cumulative probability.
(0.05 MB PNG)

Figure S7 No obvious impact of the eQTN location on the mapping precision. Cumulative plot of the distance between the most significant SNPs and the actual eQTNs according to the eQTN location (upstream of the TSS, downstream of the TSS, within an exon, and within an intron). This plot was generated by averaging results from the p-value method applied to 10 simulated dataset (see Text S1). For the legend, the percentage between brackets give the fraction of actual eQTNs in the corresponding category.
(0.08 MB PNG)

Figure S8 Impact of the local recombination rate on the eQTN mapping precision. Boxplot of the physical distance between the tag SNP and the actual eQTN as a function of the average recombination rate (cM/Mb) around the actual eQTN in a simulated dataset assuming that all eQTNs are not genotyped (see Text S1). We divided the data into four categories of equal sizes (from low to high level of recombination rate, the range of the recombination rate in each class is indicated along the x-axis below each box). As expected, the higher the recombination rate, the lower the expected distance between the tag SNP and the actual eQTN.
(0.05 MB PNG)

Figure S9 There is a deficit of most-significant SNPs in internal introns, and an enrichment of such SNPs in last exons (p-value method). This figure is based on the subset of 295 genes for which there is a unique most significant SNP (and for which the smallest p-value is <7×10^−6^) that fall into the gene transcript region. For the five panels, the blue arrows represent the observed number of most significant SNPs in the five gene functional elements for which at least 5 most significant SNPs have been found. Here these counts have been corrected for putative spurious signal due to an unobserved SNP inside the probe (leading to the removal of {similar, tilde operator } 46 genes). Under the null hypothesis that these most significant SNPs are randomly distributed into the eight possible gene functional elements, we carried out a simple Monte-Carlo procedure where for each of the 295 genes we picked at random a SNP inside the gene transcript region to be the most significant SNP (and weight it by the probability that the gene has genuine signal according to the location of the observed most significant SNP with respect to the probe (see Text S1). The histograms depict the distribution of the numbers of most significant SNPs across 1000 simulated configurations.
(0.12 MB PNG)

Figure S10 When distance is measured from the TSS (or TES) only, the TES (or TSS) peak is hidden due to the great variability in gene lengths. The plots show the fraction of SNPs with eQTN signals as a function of position in the *cis*-candidate region. The candidate region is divided into a series of 1 kb bins across the x-axis that indicate position relative to the TSS (or TES). For each bin we plot the proportion of SNPs that have the smallest p-value for the corresponding gene, and for which p<7×10^−6^ (gene FDR of 5%).
(0.07 MB PNG)

Figure S11 Illustration of the ability of the HM to accurately estimate the distribution of eQTNs even when only 30% of the actual eQTNs are genotyped. These plots are based on a simulated dataset assuming that across all genes only 30% of the true eQTNs are genotyped (see Text S1). In both panels the black histograms represent the number of actual eQTNs using 1 kb bins anchored from the TSS (this is identical for both panels). A. P-value method: the green curve displays the number of most significant SNPs detected by the p-value method. As expected, due to the uncomplete SNP coverage, LD and the stringency of the p-value cut-off, the profile is less peaked than the actual distribution. B. Hierarchical model (TSS-only version): the red curve indicates the expected number of eQTNs computed using the posterior probabilities from the hierarchical model. The hierarchical model provides us with a much more accurate representation of the actual eQTN distribution.
(0.20 MB PNG)

Figure S12 Simulated dataset with eQTNs symmetrically distributed around the TSS. The three left panels plot the true (simulated) probability to be the actual eQTN according to the gene size category. The three right panels plot the probability to be the most significant SNP (i.e the SNP with the smallest p-value inside the *cis*-candidate region) in genes having at least one SNP with a p-value lower than 7×10^−6^ (as for Figure 2 in the main text). Although only 30% of the actual eQTNs are observed, the distribution of the most significant SNPs (right panels) lines up pretty well with the distribution of the actual eQTNs (left panels). Furthermore, the distribution of signals for this TSS-only model is quite different than seen in the real data, consistent with our results that the TSS-only model does not provide a good description of the data. See Text S1 for a description of our simulation process.
(0.43 MB PNG)

Figure S13 Numbers of SNPs inside each of the 9 mutually exclusive gene-related annotations as a function of position within the gene. SNPs inside coding exon are classified into synonymous and non-synonymous SNPs. Notice that ∼84% of genic SNPs occur inside internal introns.
(0.12 MB PNG)

Figure S14 Fine-scale structure of eQTN peaks near the TSS and TES, and comparison to four types of functional annotation. The left- and right-hand columns show data for 5 kb on either side of the TSS and TES, respectively (averaging across all gene sizes). Locations inside genes are colored green and outside genes are black. A. Posterior expected fractions of SNPs in each bin that are eQTNs, as estimated by the hierarchical model (see Methods). Each bin is 25 bp wide. B. Probability that a SNP falls into a (putative) functional site: CpG island (CpG), conserved non-coding element (CNC), predicted *cis*-regulatory module (pCRM) and micro RNA binding site (miRNA).
(0.27 MB PNG)

Figure S15 Genes with CpG islands spanning the TSS are expressed at higher average levels and are more likely to contain eQTLs than genes without a CpG island at the TSS. Results for genes with a CpG island ON the TSS are displayed in red while results for genes without a CpG island spanning the TSS (OFF) are displayed in black. These results are based by computing seperately for the two gene categories the posterior probabilities from the hierarchical model. A. Estimated probability for each gene category to have an eQTN anywhere in the *cis*-candidate region. B. Box plots of the means and the standard deviations of the log hybridization intensities for the two gene categories. Genes ON CpG have higher mean expression and standard deviations than Gene OFF CpG. C. After adjusting for the different overall rates of eQTNs, the distribution of signal locations in the two classes of genes is very similar. The plots show the fraction of SNPs with eQTN signals as a function of position in the *cis*-candidate region, based on the hierarchical model. In order to make the two classes of genes more comparable, the plots are conditional on the gene having an eQTN. Top panel shows results for the 7,069 genes with a CpG island spanning the TSS (ON CpG) and bottom panel shows results for the 4,377 genes without a CpG island spanning the TSS (OFF CpG).
(0.27 MB PNG)

Figure S16 Schematic explanations of our gene structure annotation. The plot shows three pairs of hypothetical genes consisting of, respectively, 1, 2 and 6 exons. In each pair, the upper version of the gene shows the exon/intron structure (from RefSeq) and the translation start and stop sites (vertical red lines). The lower version of the gene shows how we annotate the gene structure (see color code at right of figure). A verbal explanation is also provided in the main text.
(0.17 MB PNG)

Figure S17 Locations of the most significant eQTL SNPs for small, medium, and large genes using a p-value cutoff of A) 1×10^−2^ and B) 1×10^−4^. For A and B, the three panels was prepared in the same way as Figure 2 of the main paper.
(0.44 MB PNG)

Figure S18 Locations of the most significant eQTL SNPs for small, medium, and large genes using a p-value cutoff of A) 1×10^−6^ and B) 1×10^−8^. For A and B, the three panels was prepared in the same way as Figure 2 of the main paper.
(0.41 MB PNG)

Figure S19 Distribution of most significant eQTL SNPs around probes. The black bars indicate the numbers of spurious eQTL signals as a function of distance from the probes, among the 634 genes with a known SNP in the probe. The sum of the red+green bars gives the numbers of most significant eQTL SNPs among the remaining 11,446 genes; the red component is our estimate of the fraction that is spurious. (See section ‘Spurious Signal’ in Text S1 for further description.)
(0.18 MB PNG)

Table S1 Table of descriptive statistics for each of the 9 mutually exclusive gene structure annotations for the 11,446 genes of our data set. The “Exp nber” and “Fraction” columns of the table are based on the posterior probabilities to be a genuine eQTN from the hierarchical model: left side for TSS-only+annotation model and right side for TSS+TES+annotation model.
(0.03 MB PDF)

Table S2 Table of descriptive statistics for each of the 8 mutually exclusive gene structure annotations for the 11,446 genes of our data set.
(0.03 MB PDF)

Table S3 Table of descriptive statistics for each of the 5 functional annotations for the 11,446 genes of our data set.
(0.04 MB PDF)

Text S1 Supplementary methods.
(0.15 MB PDF)
