# Supplementary figures and images for: High-Resolution Mapping of Expression-QTLs Yields Insight into Human Gene Regulation
Source: PLoS Genet. 2008 Oct 10;4(10):e1000214. doi: 10.1371/journal.pgen.1000214 (PMC2556086; doi:10.1371/journal.pgen.1000214)

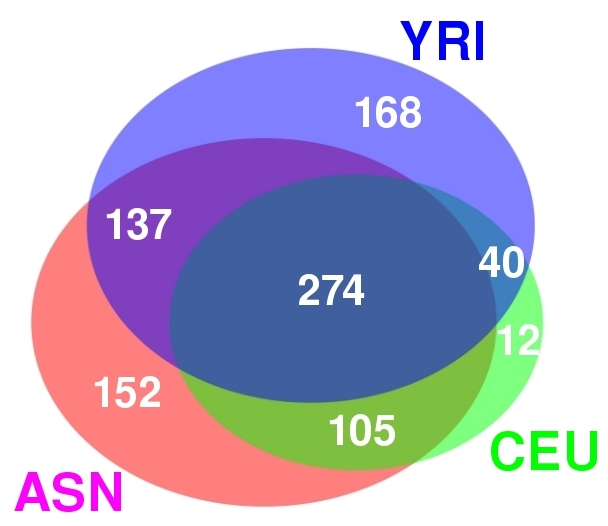

Supplement: Figure S1 — About 60% of the eQTNs are shared between at least two populations. Venn diagram of the set of eQTNs detected separately in each population. To generate the diagram, we admitted a SNP to the analysis (as an eQTL) if either the p-value in the combined sample (pooling the 3 populations) is lower than 7×10^−6^ or the p-value in a single population is lower than the p-value cutoff corresponding to a gene FDR of 5% within each population. We then considered two populations to share an eQTL if any single population has a p-value <1×10^−2^. Finally, for each gene having at least one such eQTL, we defined the eQTN as the SNP with the largest number of shared populations (sharing weight between multiple SNPs if there is a tie). (0.12 MB PNG) [file pgen.1000214.s001.png]

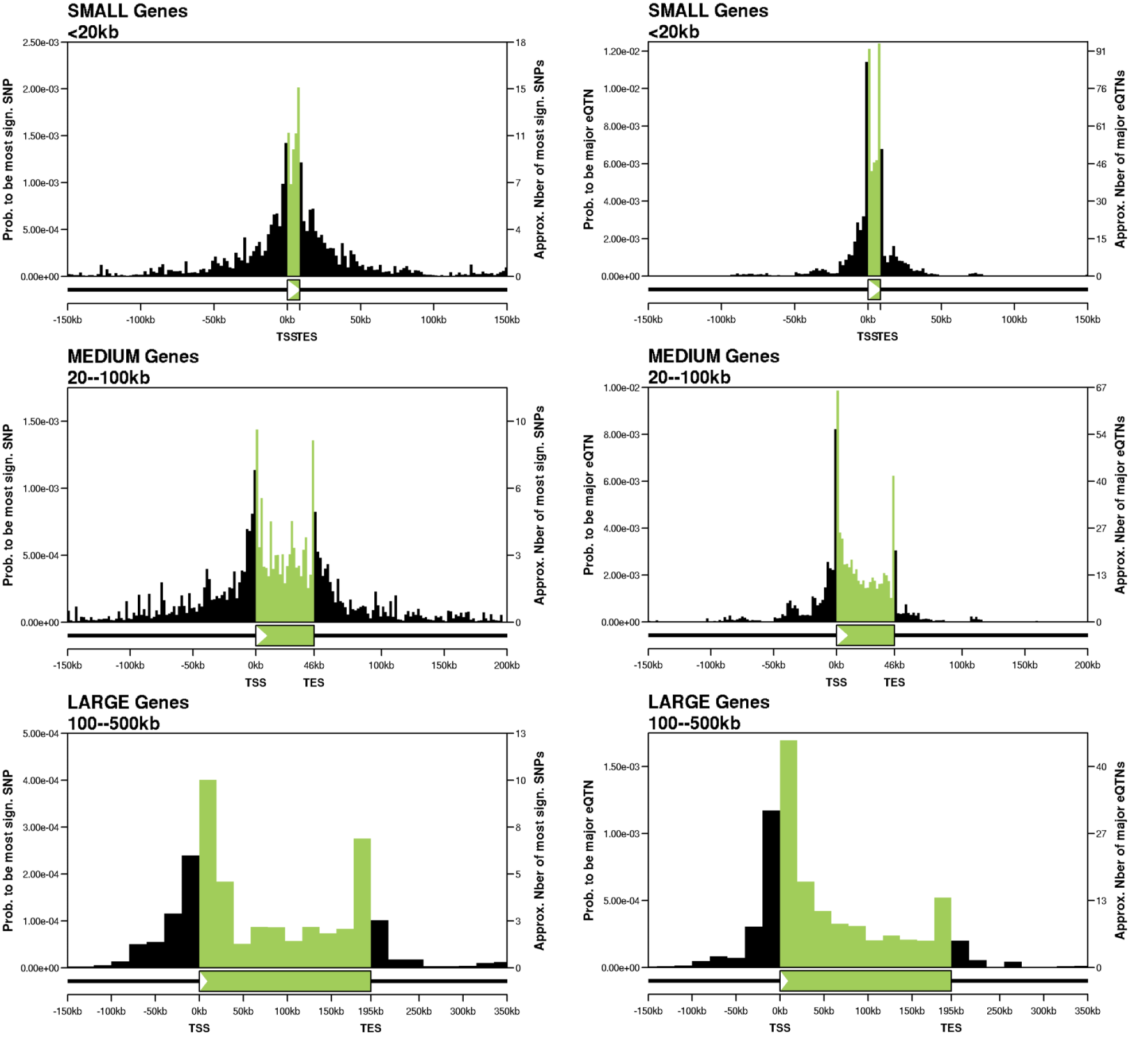

Supplement: Figure S2 — Expression QTNs in the combined Japanese plus Chinese analysis panel (ASN) show similar patterns to those in the full data. The left panel (p-value method) was prepared in the same way as Figure 2 of the main paper and the right panel (hierarchical model with TSS+TES) was prepared in the same way as Figure 3 (left panel) of the main paper. Both display results analyzing only the Asian data. For the left panels we used a p-value cutoff of 1.25×10^−5^ obtained by permutations when analyzing only the Asian data and corresponding to a gene FDR of 5%. (0.43 MB PNG) [file pgen.1000214.s002.png]

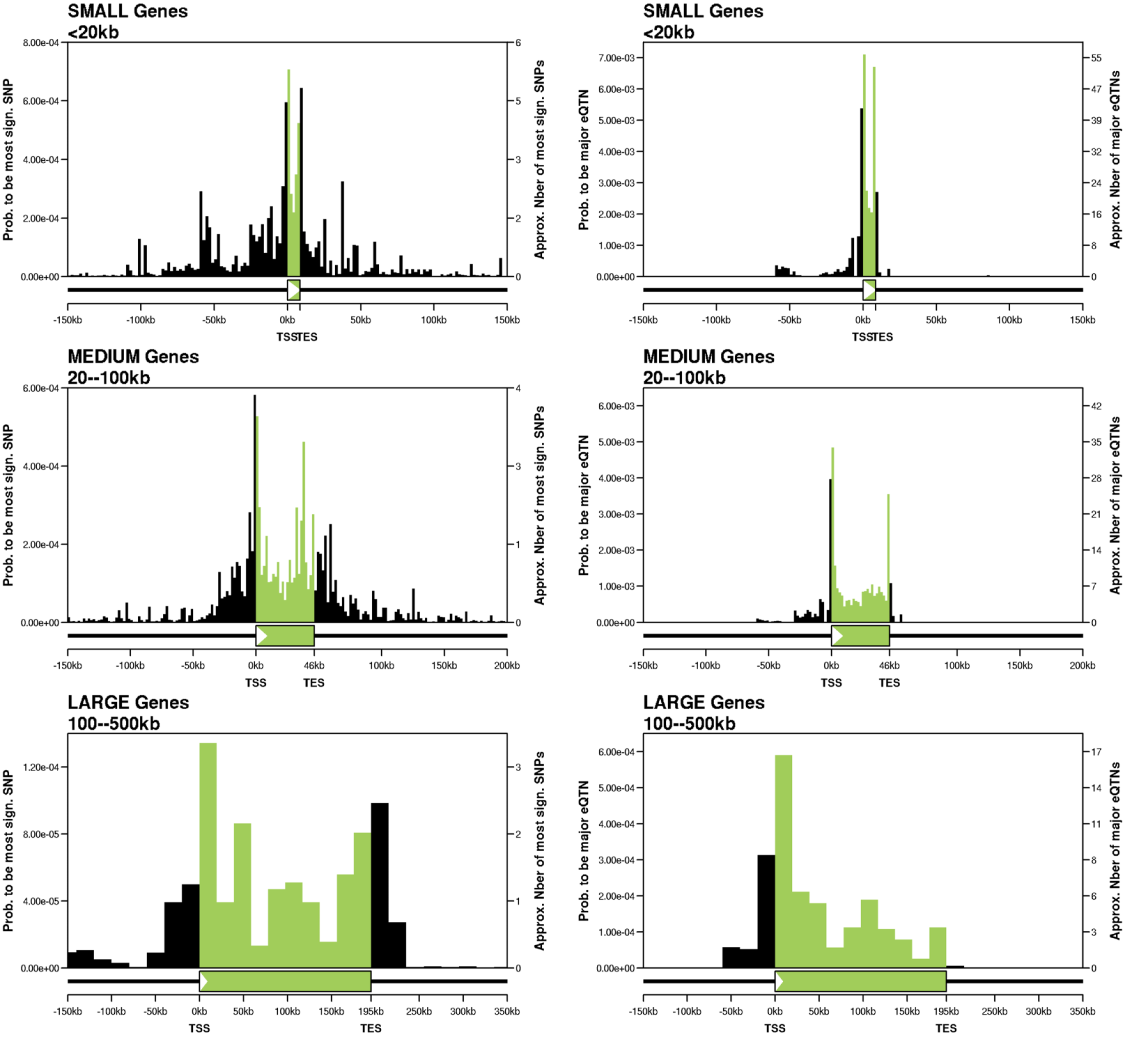

Supplement: Figure S3 — Expression QTNs in the European-derived sample (CEU) show similar patterns to those in the full data. The left panel (p-value method) was prepared in the same way as Figure 2 of the main paper and the right panel (hierarchical model with TSS+TES) was prepared in the same way as Figure 3 (left panel) of the main paper. Both display results analyzing only the European data. For the left panels we used a p-value cutoff of 3.5×10^−6^ obtained by permutations when analyzing only the European data and corresponding to a gene FDR of 5%. (0.46 MB PNG) [file pgen.1000214.s003.png]

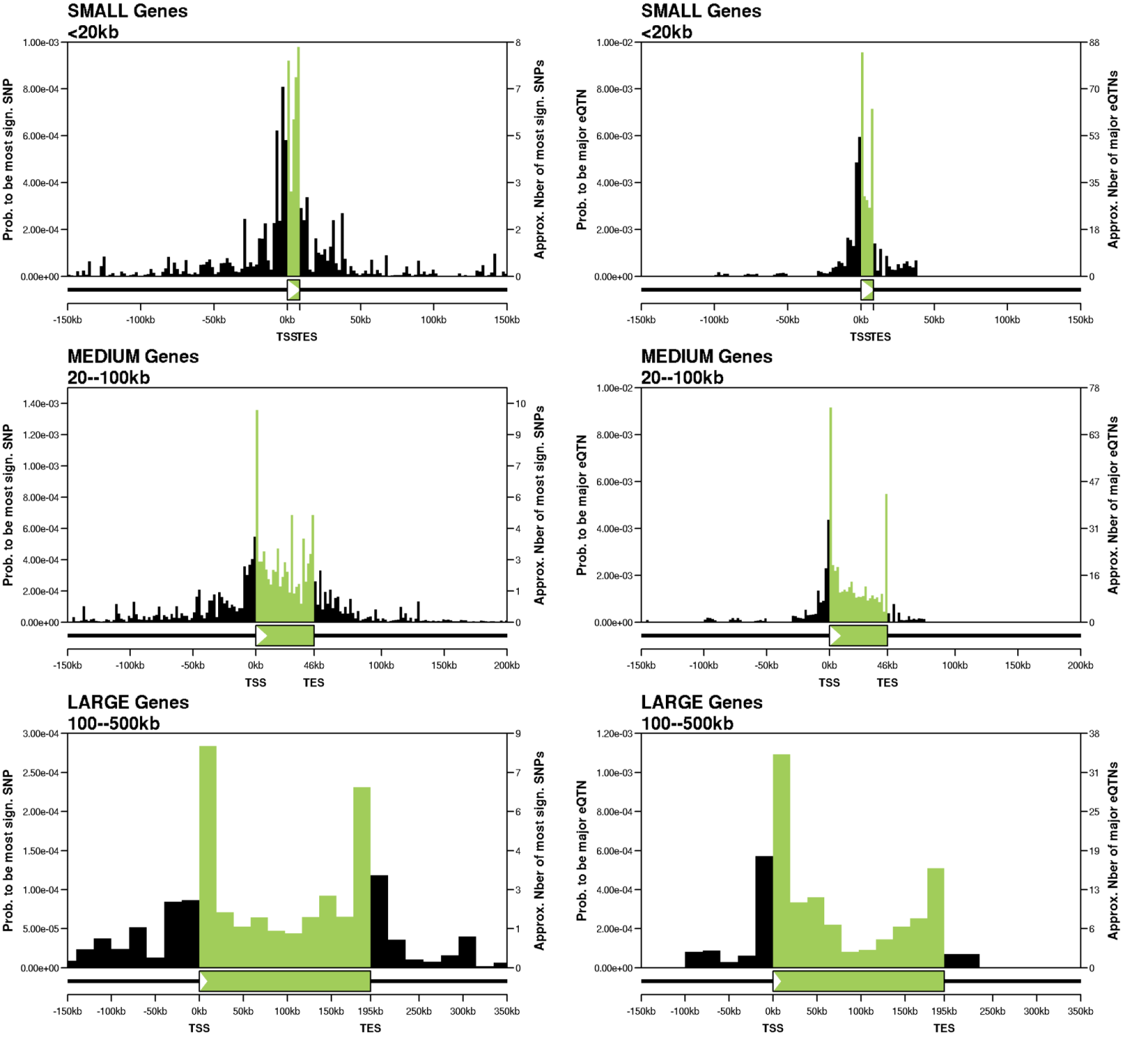

Supplement: Figure S4 — Expression QTNs in the Nigerian sample (YRI) show similar patterns to those in the full data. The left panel (p-value method) was prepared in the same way as Figure 2 of the main paper and the right panel (hierarchical model with TSS+TES) was prepared in the same way as Figure 3 (left panel) of the main paper. Both display results analyzing only the Nigerian data. For the left panels we used a p-value cutoff of 3.825×10^−6^ obtained by permutations when analyzing only the Nigerian data and corresponding to a gene FDR of 5%. (0.43 MB PNG) [file pgen.1000214.s004.png]

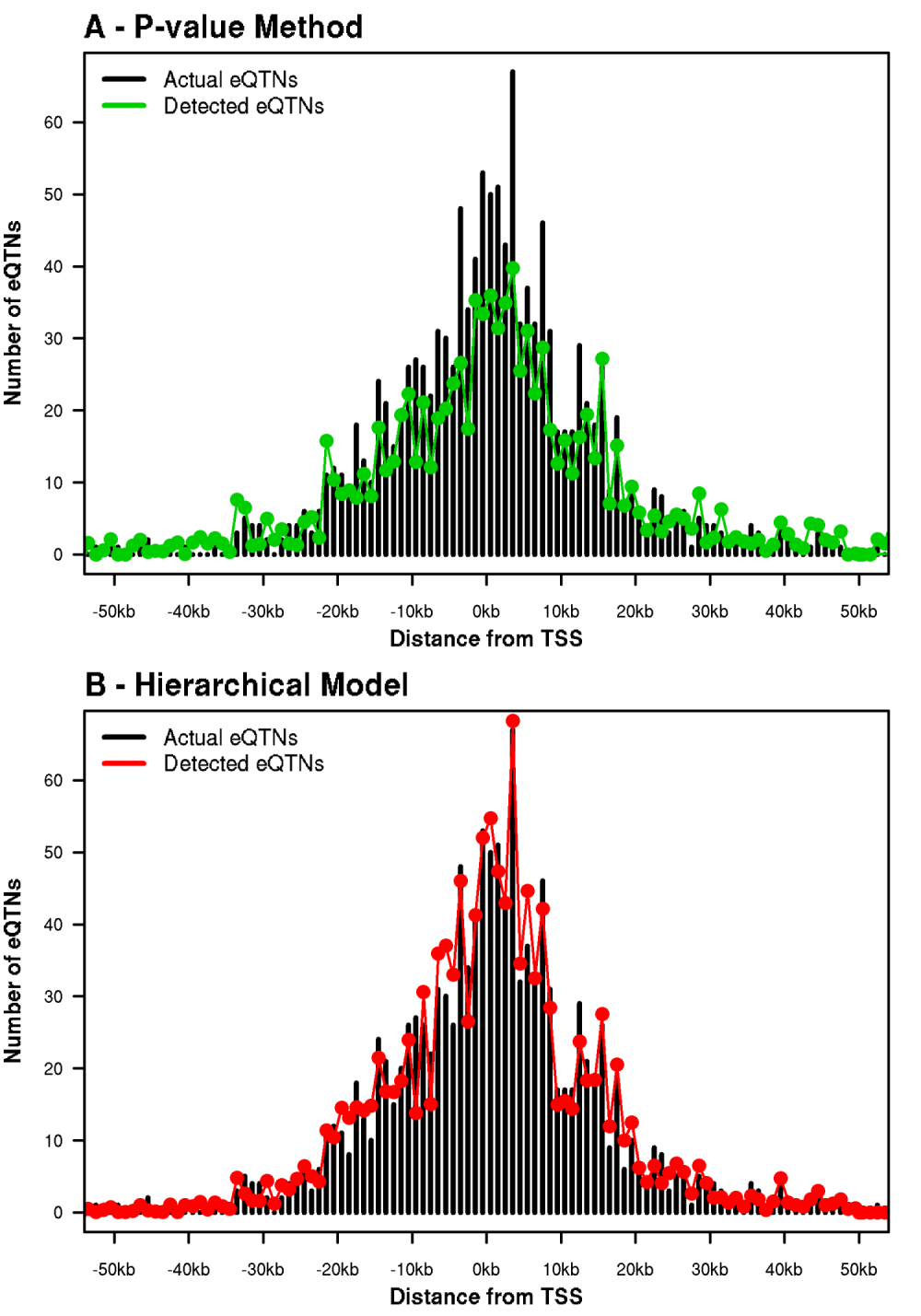

Supplement: Figure S5 — Illustration of the ability of the HM to accurately estimate the distribution of eQTNs when all the actual eQTNs are genotyped. This figure is based on a simulated dataset assuming that for all genes the actual eQTN is genotyped (see Text S1). In both panels the black histograms represent the number of actual eQTNs using 1 kb bins anchored from the TSS (this is identical for both panels). A. P-value method: the green curve displays the number of most significant SNPs detected by the p-value method. As expected, due to LD and the stringency of the p-value cut-off, the profile is less peaked than the actual distribution. B. Hierarchical model: using our hierarchical model with the TSS-only model (see Methods) we are able to catch most of the actual eQTNs. The red curve indicates the expected number of eQTNs computed using the posterior probabilities from the hierarchical model. Notice that the hierarchical model provides a better picture of the distribution of signals. (0.15 MB PNG) [file pgen.1000214.s005.png]

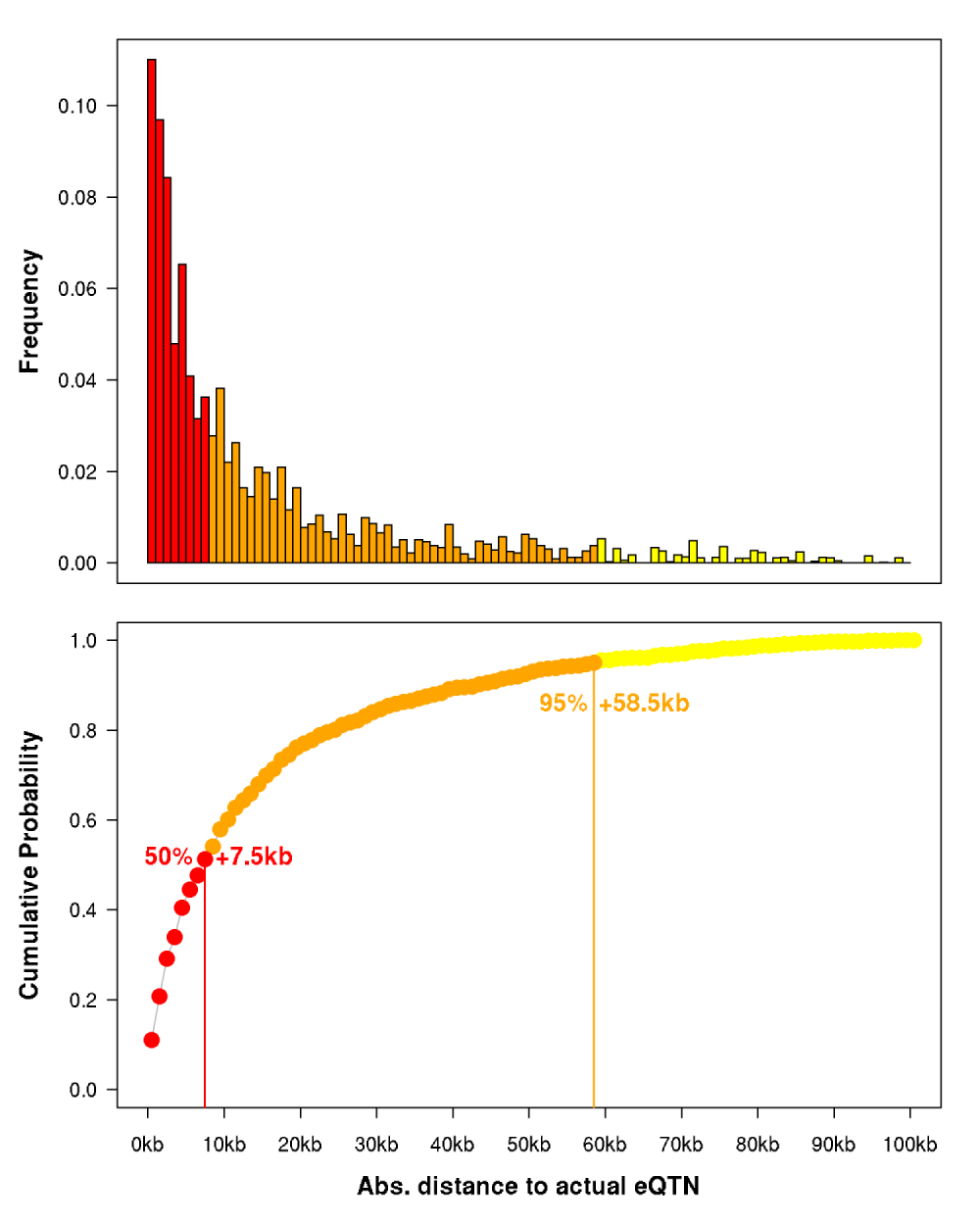

Supplement: Figure S6 — 50% of the most significant SNPs lie within 7.5 kb of the actual eQTNs. Both panels are based on the results from the p-value method applied to a simulated dataset (see Text S1). The top panel plots the histogram of the fraction of most significant SNPs as a function of distance from the actual eQTNs. The bottom panel plots the corresponding cumulative probability. (0.05 MB PNG) [file pgen.1000214.s006.png]

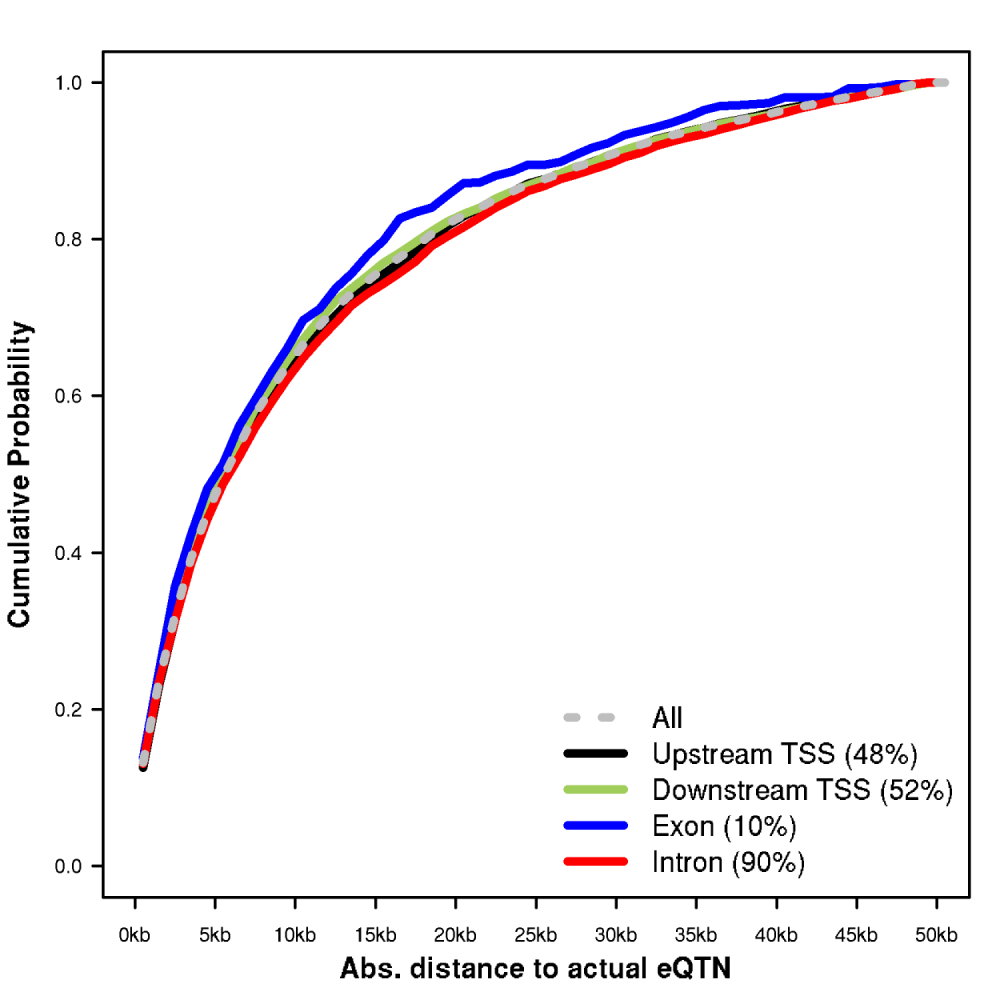

Supplement: Figure S7 — No obvious impact of the eQTN location on the mapping precision. Cumulative plot of the distance between the most significant SNPs and the actual eQTNs according to the eQTN location (upstream of the TSS, downstream of the TSS, within an exon, and within an intron). This plot was generated by averaging results from the p-value method applied to 10 simulated dataset (see Text S1). For the legend, the percentage between brackets give the fraction of actual eQTNs in the corresponding category. (0.08 MB PNG) [file pgen.1000214.s007.png]

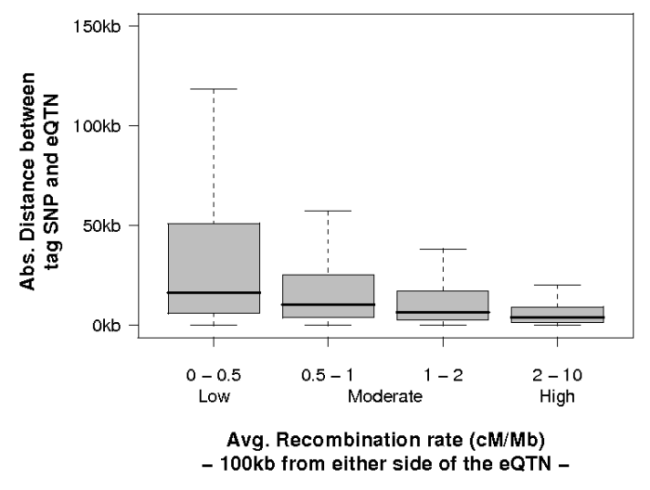

Supplement: Figure S8 — Impact of the local recombination rate on the eQTN mapping precision. Boxplot of the physical distance between the tag SNP and the actual eQTN as a function of the average recombination rate (cM/Mb) around the actual eQTN in a simulated dataset assuming that all eQTNs are not genotyped (see Text S1). We divided the data into four categories of equal sizes (from low to high level of recombination rate, the range of the recombination rate in each class is indicated along the x-axis below each box). As expected, the higher the recombination rate, the lower the expected distance between the tag SNP and the actual eQTN. (0.05 MB PNG) [file pgen.1000214.s008.png]

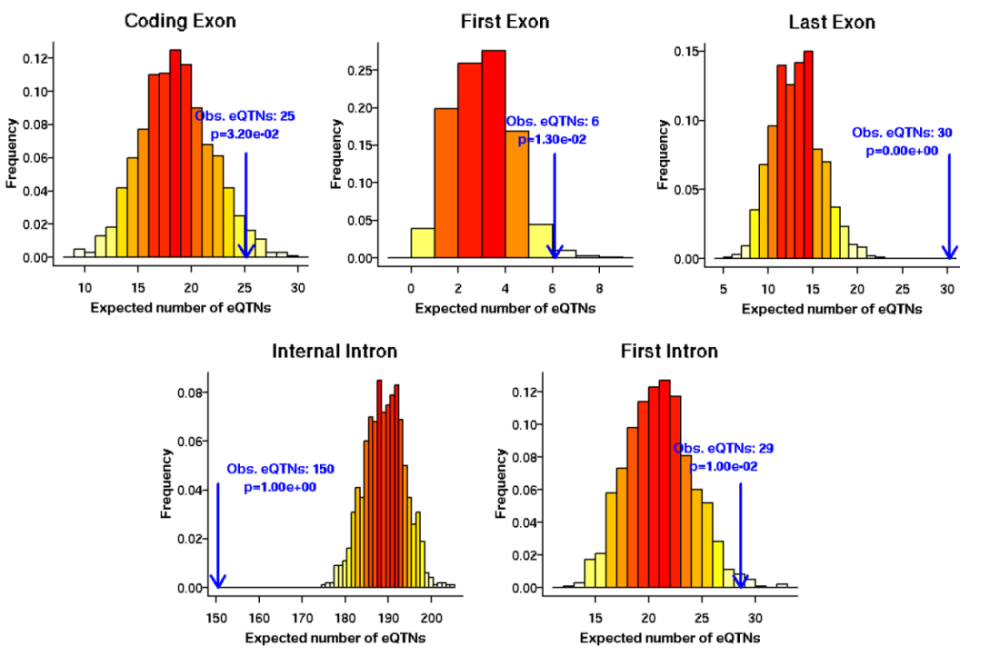

Supplement: Figure S9 — There is a deficit of most-significant SNPs in internal introns, and an enrichment of such SNPs in last exons (p-value method). This figure is based on the subset of 295 genes for which there is a unique most significant SNP (and for which the smallest p-value is <7×10^−6^) that fall into the gene transcript region. For the five panels, the blue arrows represent the observed number of most significant SNPs in the five gene functional elements for which at least 5 most significant SNPs have been found. Here these counts have been corrected for putative spurious signal due to an unobserved SNP inside the probe (leading to the removal of {similar, tilde operator } 46 genes). Under the null hypothesis that these most significant SNPs are randomly distributed into the eight possible gene functional elements, we carried out a simple Monte-Carlo procedure where for each of the 295 genes we picked at random a SNP inside the gene transcript region to be the most significant SNP (and weight it by the probability that the gene has genuine signal according to the location of the observed most significant SNP with respect to the probe (see Text S1). The histograms depict the distribution of the numbers of most significant SNPs across 1000 simulated configurations. (0.12 MB PNG) [file pgen.1000214.s009.png]

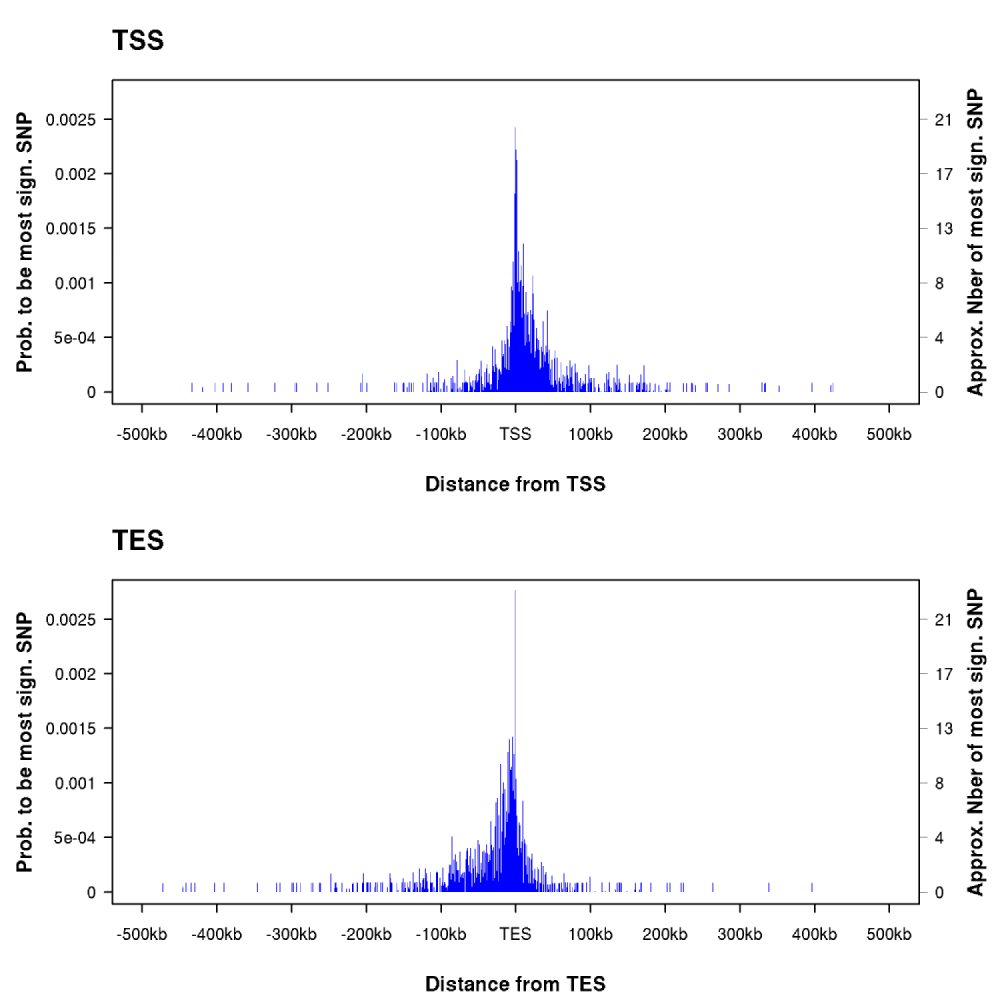

Supplement: Figure S10 — When distance is measured from the TSS (or TES) only, the TES (or TSS) peak is hidden due to the great variability in gene lengths. The plots show the fraction of SNPs with eQTN signals as a function of position in the cis-candidate region. The candidate region is divided into a series of 1 kb bins across the x-axis that indicate position relative to the TSS (or TES). For each bin we plot the proportion of SNPs that have the smallest p-value for the corresponding gene, and for which p<7×10^−6^ (gene FDR of 5%). (0.07 MB PNG) [file pgen.1000214.s010.png]

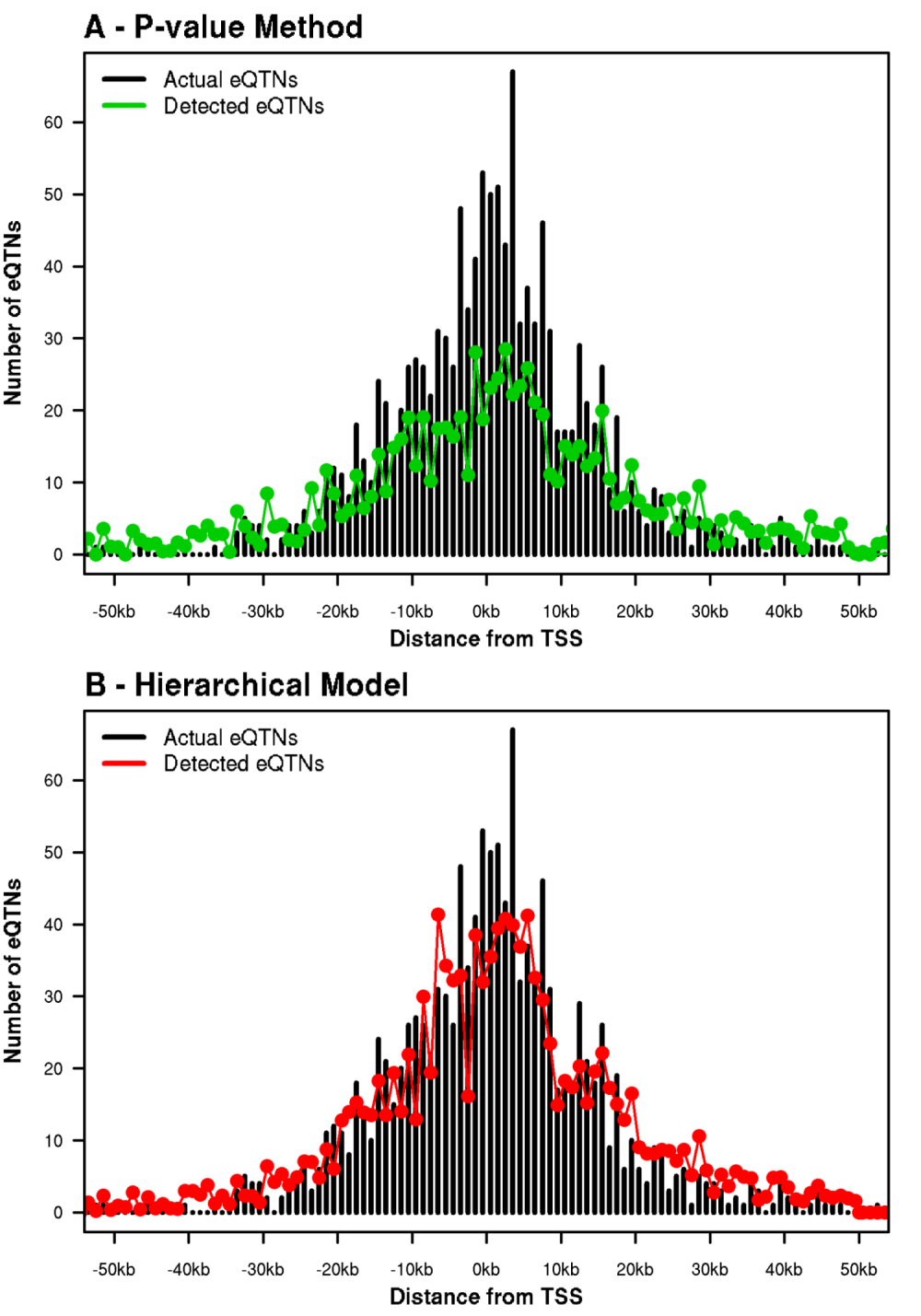

Supplement: Figure S11 — Illustration of the ability of the HM to accurately estimate the distribution of eQTNs even when only 30% of the actual eQTNs are genotyped. These plots are based on a simulated dataset assuming that across all genes only 30% of the true eQTNs are genotyped (see Text S1). In both panels the black histograms represent the number of actual eQTNs using 1 kb bins anchored from the TSS (this is identical for both panels). A. P-value method: the green curve displays the number of most significant SNPs detected by the p-value method. As expected, due to the uncomplete SNP coverage, LD and the stringency of the p-value cut-off, the profile is less peaked than the actual distribution. B. Hierarchical model (TSS-only version): the red curve indicates the expected number of eQTNs computed using the posterior probabilities from the hierarchical model. The hierarchical model provides us with a much more accurate representation of the actual eQTN distribution. (0.20 MB PNG) [file pgen.1000214.s011.png]

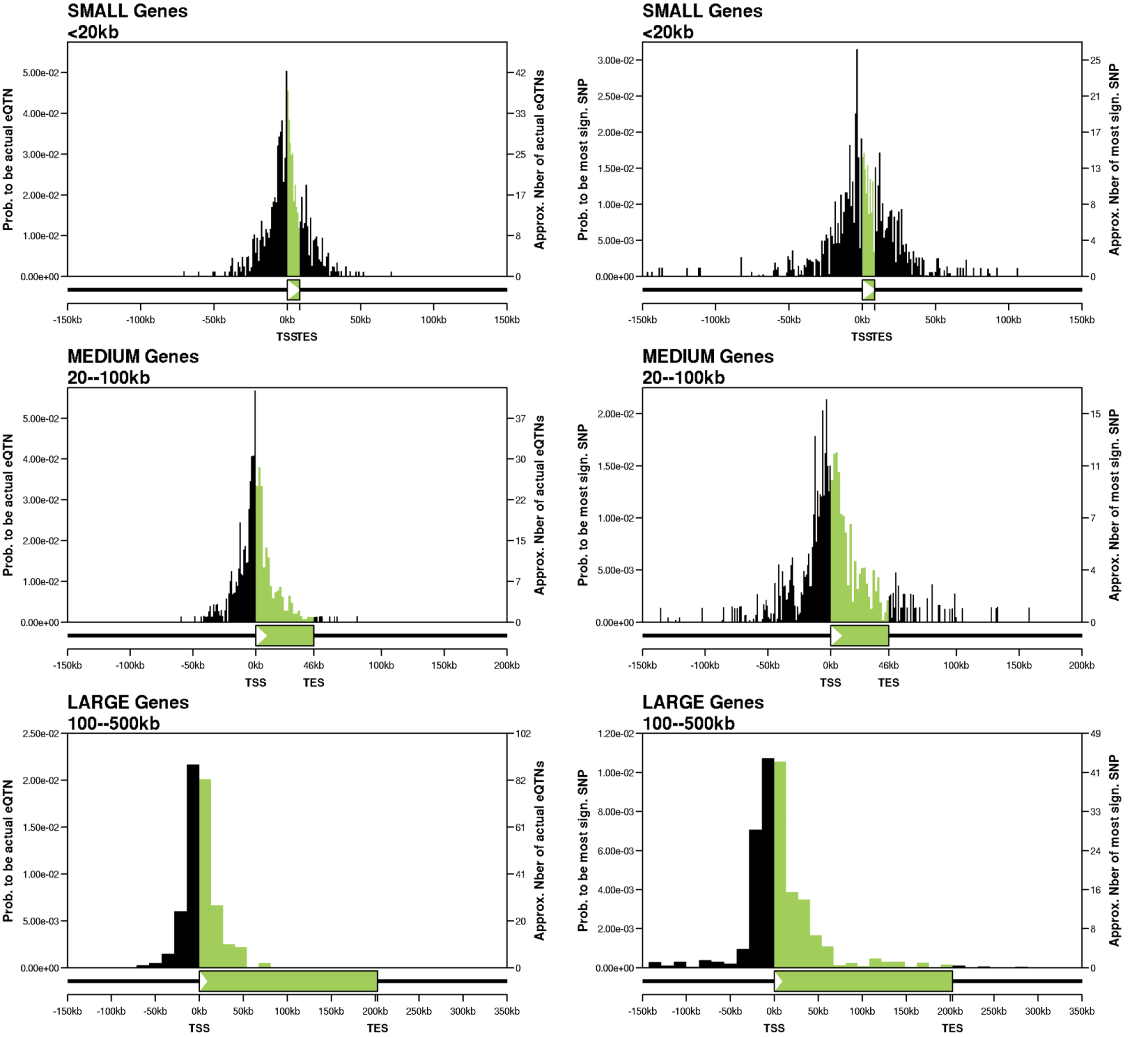

Supplement: Figure S12 — Simulated dataset with eQTNs symmetrically distributed around the TSS. The three left panels plot the true (simulated) probability to be the actual eQTN according to the gene size category. The three right panels plot the probability to be the most significant SNP (i.e the SNP with the smallest p-value inside the cis-candidate region) in genes having at least one SNP with a p-value lower than 7×10^−6^ (as for Figure 2 in the main text). Although only 30% of the actual eQTNs are observed, the distribution of the most significant SNPs (right panels) lines up pretty well with the distribution of the actual eQTNs (left panels). Furthermore, the distribution of signals for this TSS-only model is quite different than seen in the real data, consistent with our results that the TSS-only model does not provide a good description of the data. See Text S1 for a description of our simulation process. (0.43 MB PNG) [file pgen.1000214.s012.png]

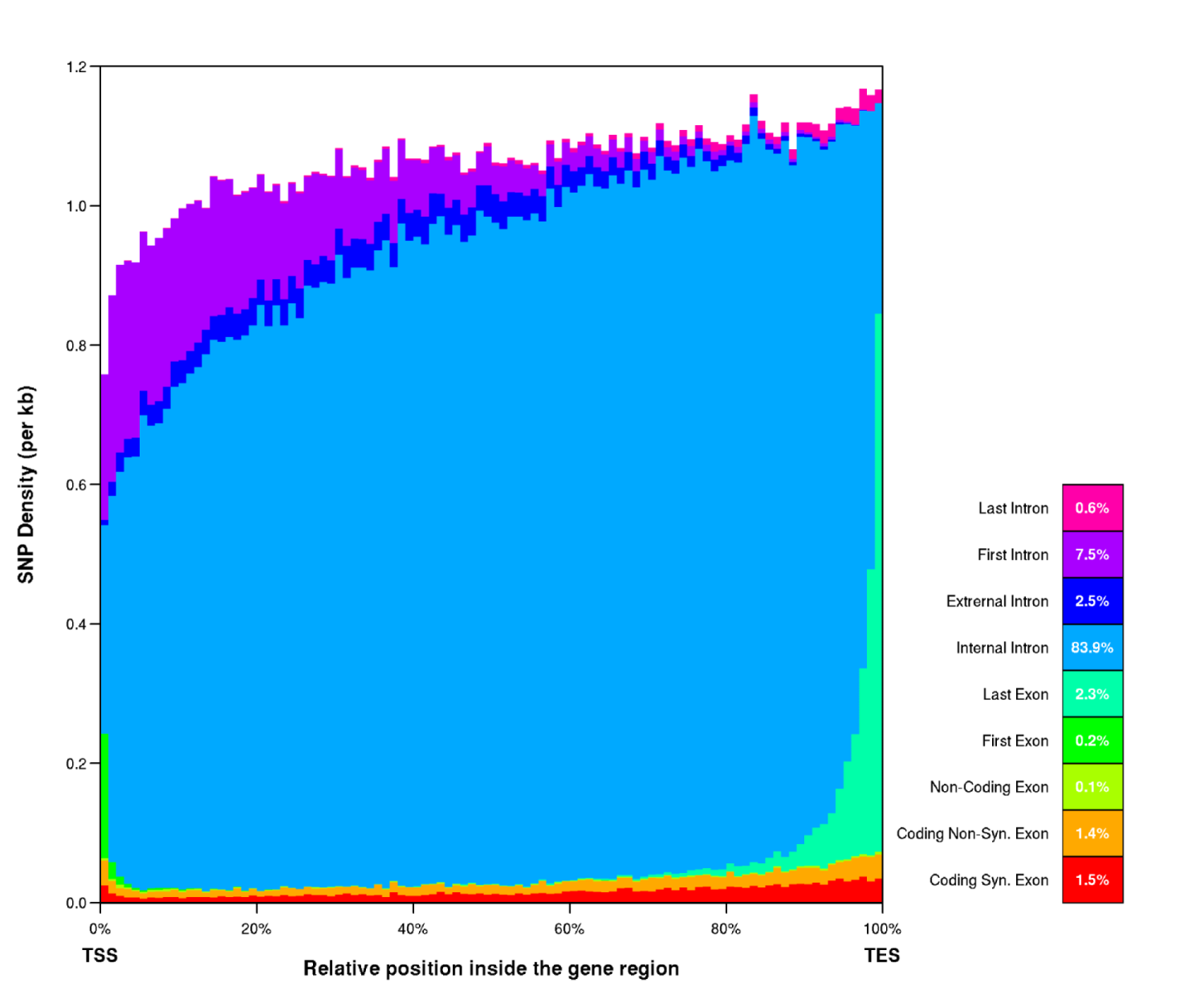

Supplement: Figure S13 — Numbers of SNPs inside each of the 9 mutually exclusive gene-related annotations as a function of position within the gene. SNPs inside coding exon are classified into synonymous and non-synonymous SNPs. Notice that ∼84% of genic SNPs occur inside internal introns. (0.12 MB PNG) [file pgen.1000214.s013.png]

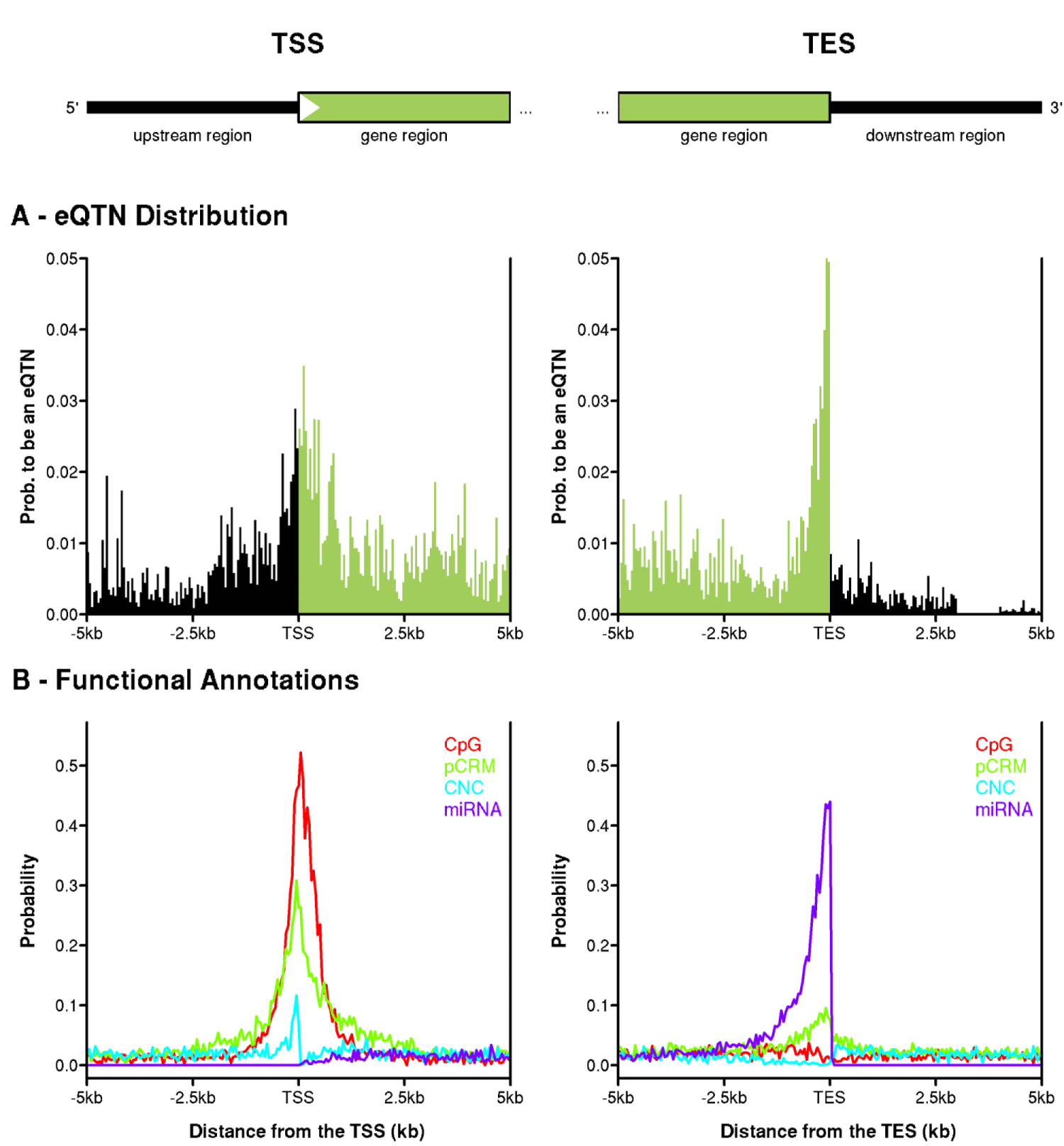

Supplement: Figure S14 — Fine-scale structure of eQTN peaks near the TSS and TES, and comparison to four types of functional annotation. The left- and right-hand columns show data for 5 kb on either side of the TSS and TES, respectively (averaging across all gene sizes). Locations inside genes are colored green and outside genes are black. A. Posterior expected fractions of SNPs in each bin that are eQTNs, as estimated by the hierarchical model (see Methods). Each bin is 25 bp wide. B. Probability that a SNP falls into a (putative) functional site: CpG island (CpG), conserved non-coding element (CNC), predicted cis-regulatory module (pCRM) and micro RNA binding site (miRNA). (0.27 MB PNG) [file pgen.1000214.s014.png]

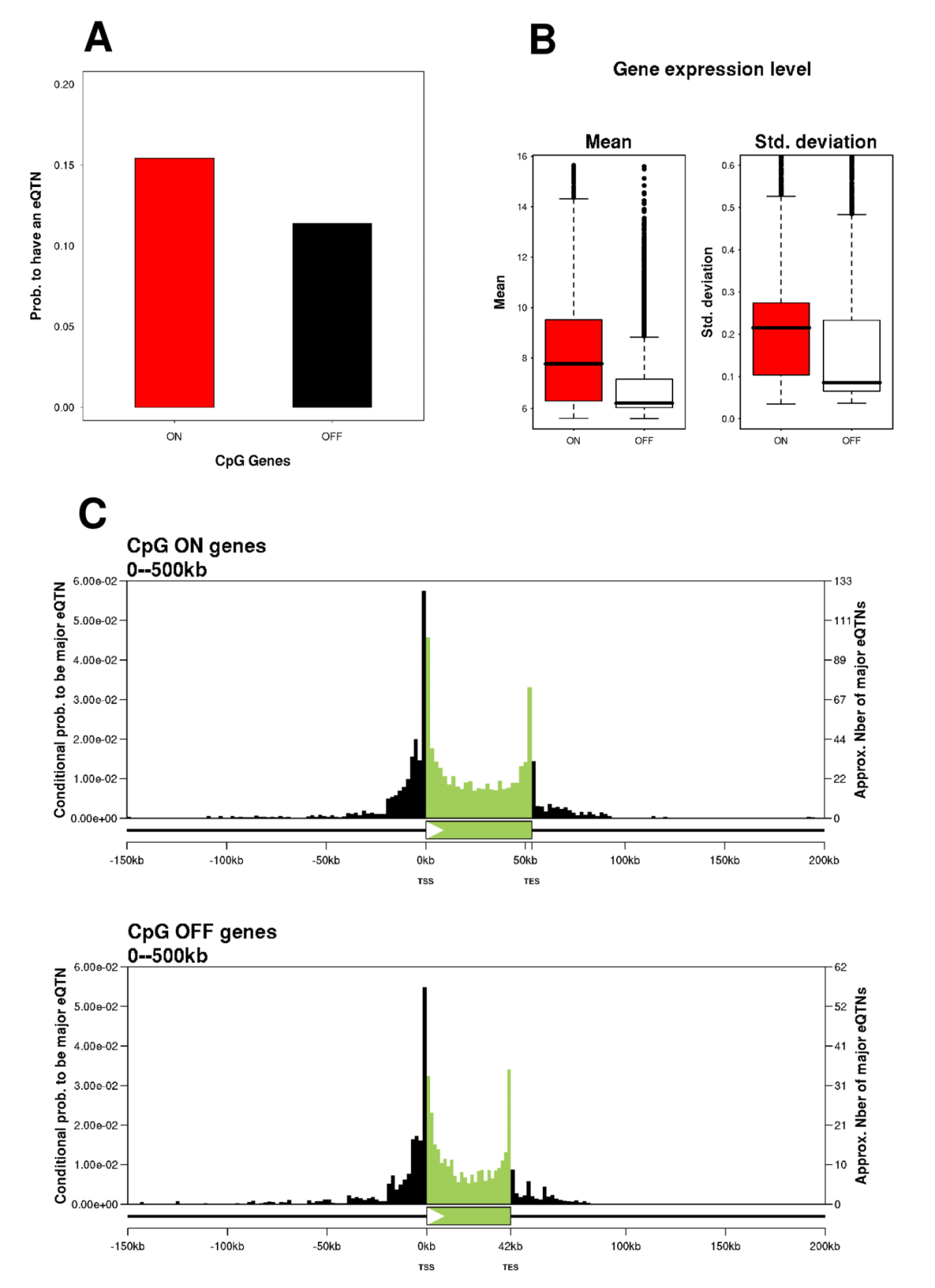

Supplement: Figure S15 — Genes with CpG islands spanning the TSS are expressed at higher average levels and are more likely to contain eQTLs than genes without a CpG island at the TSS. Results for genes with a CpG island ON the TSS are displayed in red while results for genes without a CpG island spanning the TSS (OFF) are displayed in black. These results are based by computing seperately for the two gene categories the posterior probabilities from the hierarchical model. A. Estimated probability for each gene category to have an eQTN anywhere in the cis-candidate region. B. Box plots of the means and the standard deviations of the log hybridization intensities for the two gene categories. Genes ON CpG have higher mean expression and standard deviations than Gene OFF CpG. C. After adjusting for the different overall rates of eQTNs, the distribution of signal locations in the two classes of genes is very similar. The plots show the fraction of SNPs with eQTN signals as a function of position in the cis-candidate region, based on the hierarchical model. In order to make the two classes of genes more comparable, the plots are conditional on the gene having an eQTN. Top panel shows results for the 7,069 genes with a CpG island spanning the TSS (ON CpG) and bottom panel shows results for the 4,377 genes without a CpG island spanning the TSS (OFF CpG). (0.27 MB PNG) [file pgen.1000214.s015.png]

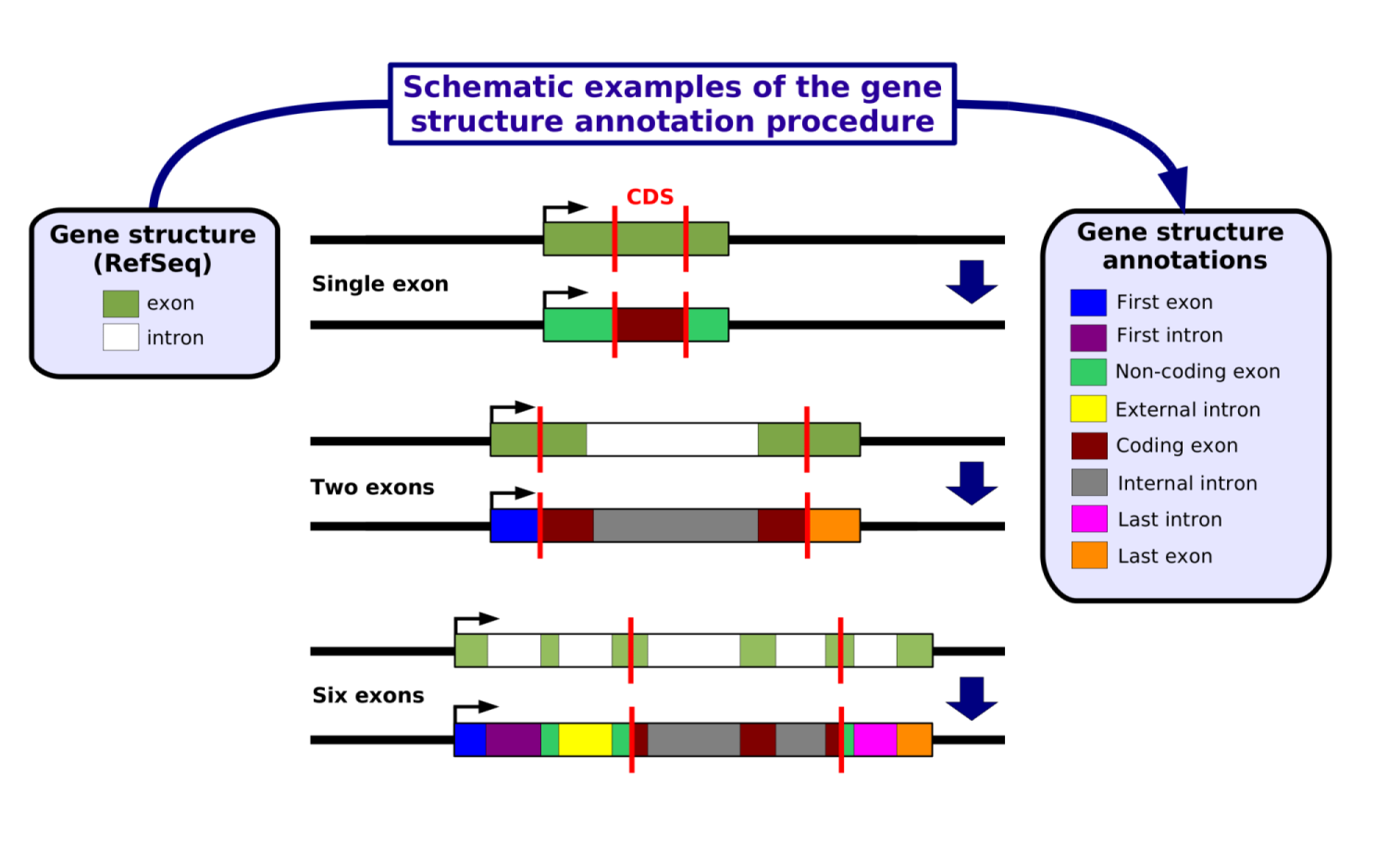

Supplement: Figure S16 — Schematic explanations of our gene structure annotation. The plot shows three pairs of hypothetical genes consisting of, respectively, 1, 2 and 6 exons. In each pair, the upper version of the gene shows the exon/intron structure (from RefSeq) and the translation start and stop sites (vertical red lines). The lower version of the gene shows how we annotate the gene structure (see color code at right of figure). A verbal explanation is also provided in the main text. (0.17 MB PNG) [file pgen.1000214.s016.png]

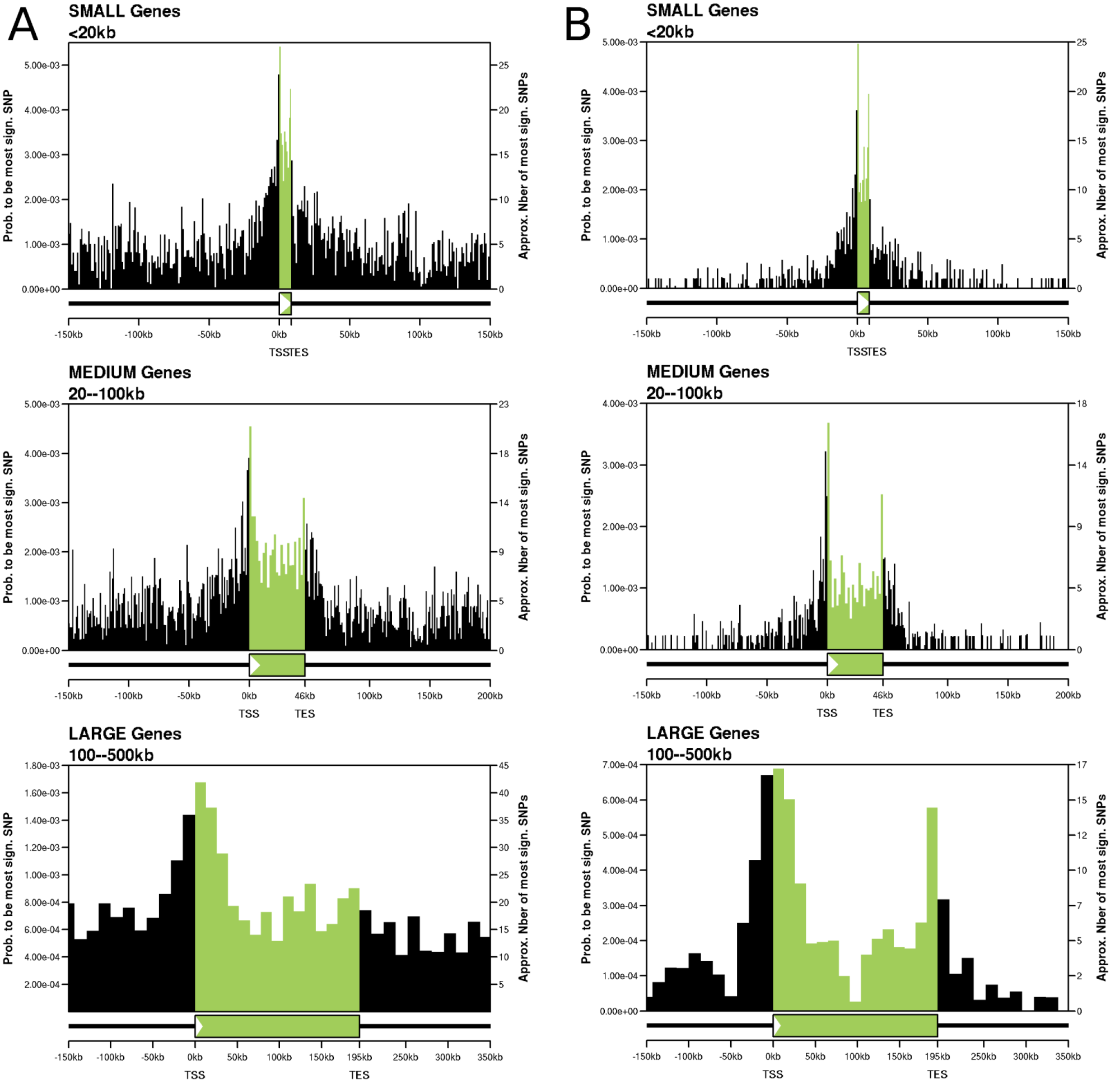

Supplement: Figure S17 — Locations of the most significant eQTL SNPs for small, medium, and large genes using a p-value cutoff of A) 1×10^−2^ and B) 1×10^−4^. For A and B, the three panels was prepared in the same way as Figure 2 of the main paper. (0.44 MB PNG) [file pgen.1000214.s017.png]

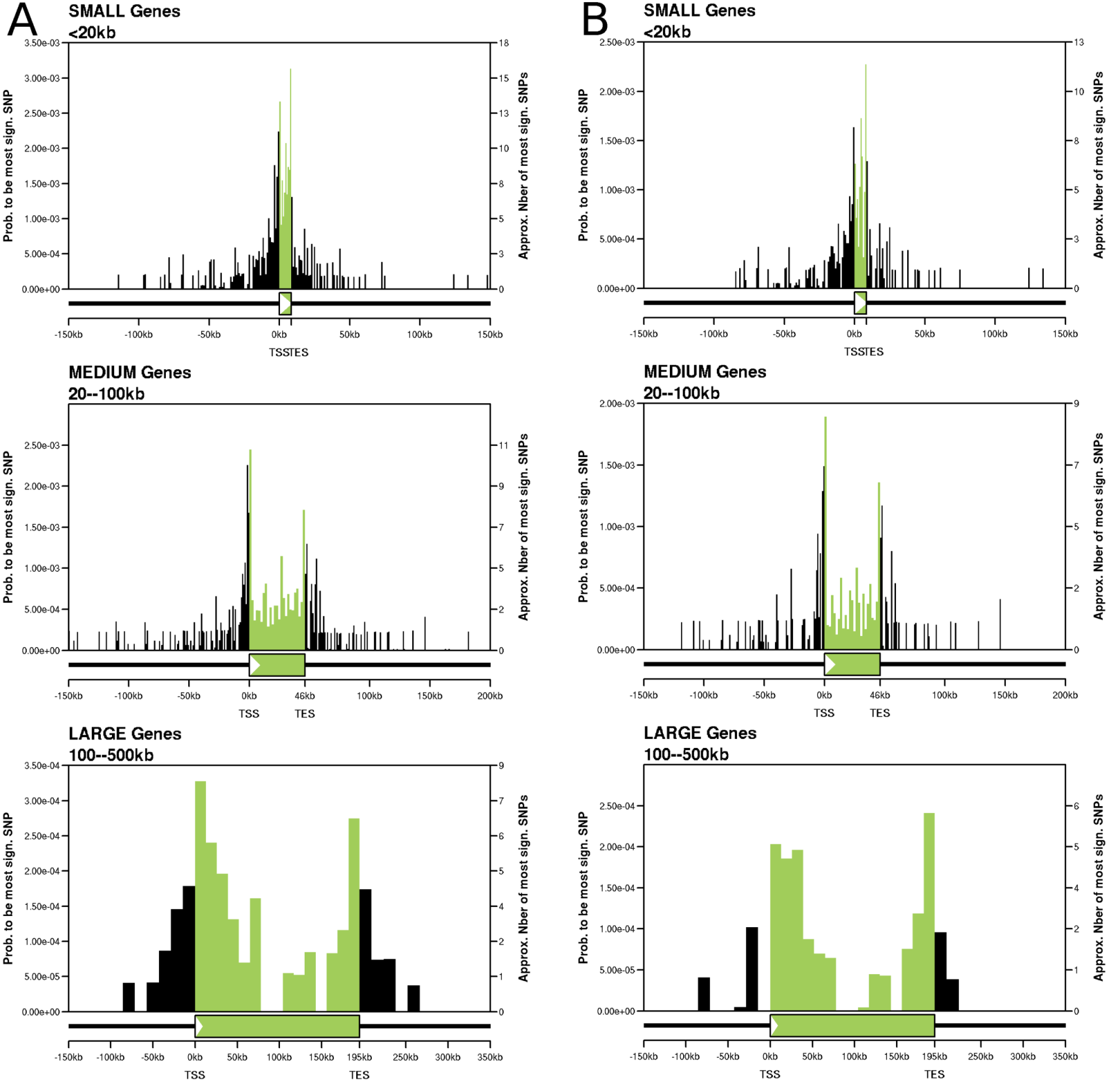

Supplement: Figure S18 — Locations of the most significant eQTL SNPs for small, medium, and large genes using a p-value cutoff of A) 1×10^−6^ and B) 1×10^−8^. For A and B, the three panels was prepared in the same way as Figure 2 of the main paper. (0.41 MB PNG) [file pgen.1000214.s018.png]

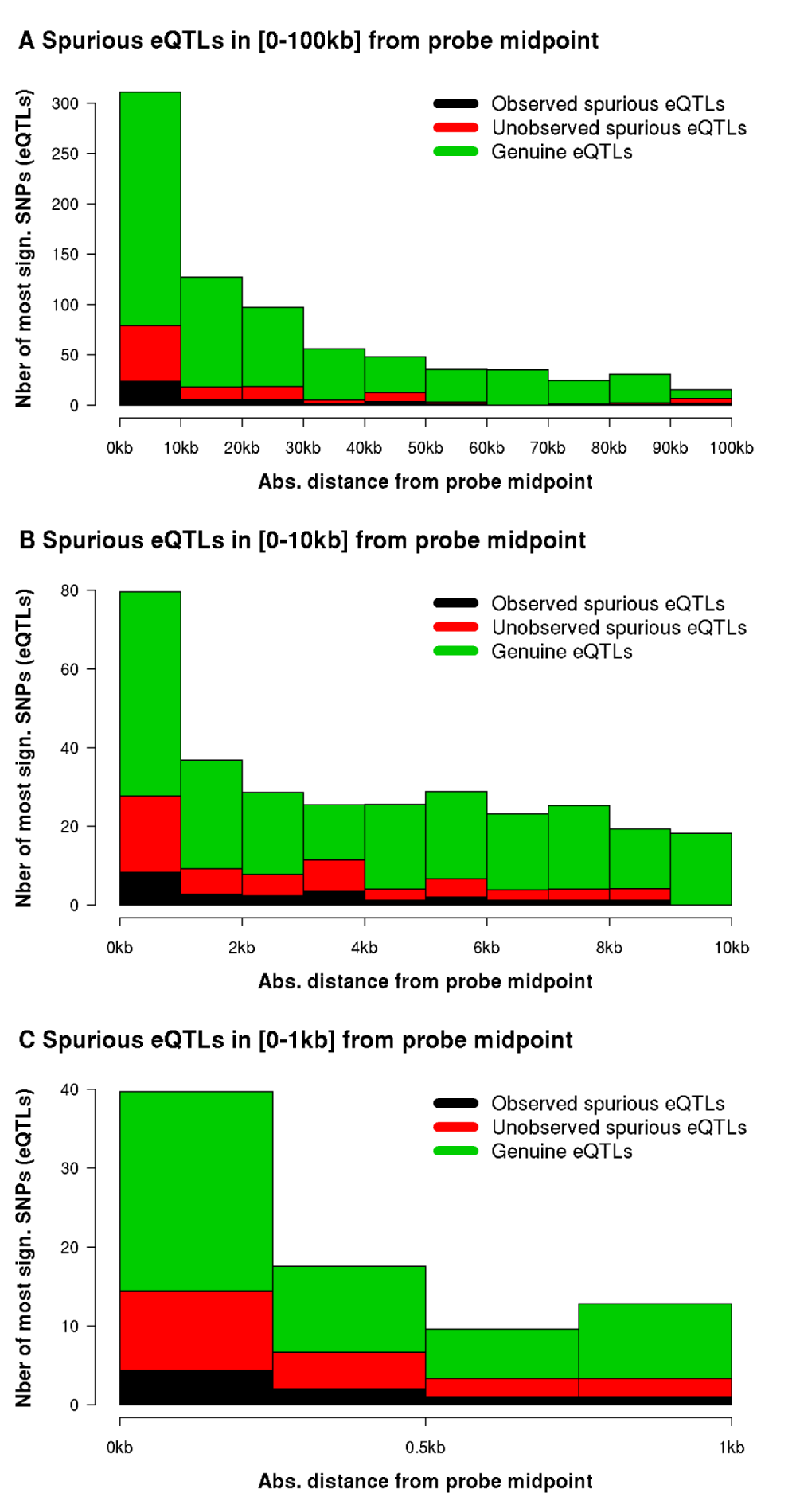

Supplement: Figure S19 — Distribution of most significant eQTL SNPs around probes. The black bars indicate the numbers of spurious eQTL signals as a function of distance from the probes, among the 634 genes with a known SNP in the probe. The sum of the red+green bars gives the numbers of most significant eQTL SNPs among the remaining 11,446 genes; the red component is our estimate of the fraction that is spurious. (See section ‘Spurious Signal’ in Text S1 for further description.) (0.18 MB PNG) [file pgen.1000214.s019.png]
