# Supplementary material for: High-Resolution Mapping of Expression-QTLs Yields Insight into Human Gene Regulation
Source: PLoS Genet. 2008 Oct 10;4(10):e1000214. doi: 10.1371/journal.pgen.1000214 (PMC2556086; doi:10.1371/journal.pgen.1000214)
Supplement: Table S1 — Table of descriptive statistics for each of the 9 mutually exclusive gene structure annotations for the 11,446 genes of our data set. The “Exp nber” and “Fraction” columns of the table are based on the posterior probabilities to be a genuine eQTN from the hierarchical model: left side for TSS-only+annotation model and right side for TSS+TES+annotation model. (0.03 MB PDF) [file pgen.1000214.s020.pdf]

Category	Name	TSS Model					TSS+TES Model				
		eQTNs		Odds Ratio			eQTNs		Odds Ratio		
		Exp. nber	Fraction (%)	Estimate	CI(95%)		Exp. nber	Fraction (%)	Estimate	CI(95%)	
Exon	Coding Syn.	36	4.22	1.78	0.81	3.18	46	4.72	1.94	1.00	3.18
	Coding Non-Syn.	46	5.37	2.38	1.20	4.01	55	5.66	2.41	1.34	3.80
	Non-Coding	11	1.33	3.52	0.66	10.25	13	1.31	3.90	0.78	10.97
	First	32	3.70	2.32	1.09	4.21	33	3.37	2.97	1.45	5.21
	Last	135	15.81	8.92	6.04	12.66	170	17.56	4.57	3.33	6.06
Intron	Internal	460	53.75	1.00	-	-	517	53.30	1.00	-	-
	External	7	0.84	0.62	0.00	2.53	1	0.15	0.13	0.00	1.92
	First	120	14.03	1.39	0.91	2.07	124	12.84	2.00	1.36	2.88
	Last	8	0.97	3.99	0.64	12.63	11	1.10	3.04	0.85	7.77
Entire Gene		858	100.00	3.56	2.98	4.42	969	100.00	1.68	1.44	2.08

Table S 1: **Table of descriptive statistics for each of the 9 mutually exclusive gene structure annotations for the 11,446 genes of our data set.** The "Exp nber" and "Fraction" columns of the table are based on the posterior probabilities to be a genuine eQTN from the hierarchical model: left side for TSS-only+annotation model and right side for TSS+TES+annotation model.
