# Supplementary material for: High-Resolution Mapping of Expression-QTLs Yields Insight into Human Gene Regulation
Source: PLoS Genet. 2008 Oct 10;4(10):e1000214. doi: 10.1371/journal.pgen.1000214 (PMC2556086; doi:10.1371/journal.pgen.1000214)
Supplement: Table S2 — Table of descriptive statistics for each of the 8 mutually exclusive gene structure annotations for the 11,446 genes of our data set. (0.03 MB PDF) [file pgen.1000214.s021.pdf]

Category	Name	Freq. (%)	Avg. size (kb)	Fraction of gene (%)	SNP density (kb^{-1})	Fraction of SNPs (%)	Nber of eQTLs	Fraction of eQTLs (%)
Exon	Coding	100.00	1.7	3.43	0.98	2.99	29	9.32
	Non-Coding	41.32	0.2	0.18	0.97	0.15	2	0.56
	First	93.12	0.2	0.31	0.84	0.23	7	2.32
	Last	94.47	1.1	2.11	1.23	2.29	33	10.76
Intron	Internal	91.11	44.5	82.69	1.15	83.76	198	64.15
	External	8.83	16.0	2.87	1.00	2.53	3	1.02
	First	34.98	11.3	8.07	1.05	7.47	33	10.62
	Last	4.67	3.4	0.33	2.05	0.60	4	1.25

Table S 2: **Table of descriptive statistics for each of the 8 mutually exclusive gene structure annotations for the 11,446 genes of our data set.**
