# Supplementary material for: High-Resolution Mapping of Expression-QTLs Yields Insight into Human Gene Regulation
Source: PLoS Genet. 2008 Oct 10;4(10):e1000214. doi: 10.1371/journal.pgen.1000214 (PMC2556086; doi:10.1371/journal.pgen.1000214)
Supplement: Table S3 — Table of descriptive statistics for each of the 5 functional annotations for the 11,446 genes of our data set. (0.04 MB PDF) [file pgen.1000214.s022.pdf]

Region	Annotation	SNPs	P-value method		Hierarchical Model				
			Most sig. SNPs		eQTNs		Odds Ratio		
		Fraction(%)	Number	Fraction (%)	Exp. nber	Fraction (%)	Estimate	CI(95%)	
Whole *cis*-region (100kb)	CpG	1.06	26	3.72	113	7.21	1.43	0.97	2.01
	CNC	1.92	20	2.89	42	2.65	1.92	0.95	3.28
	pCRM	1.93	23	3.21	83	5.31	1.27	0.79	1.91
	CTFC	11.28	32	4.53	42	2.68	0.96	0.51	1.61
	miRNA	0.23	13	1.87	75	4.79	1.39	0.88	2.08
TSS [-2.5kb,+2.5kb]	**CpG**	0.16 (9.40)	15	2.08 (15.23)	93	6.43 (26.16)	**1.69**	**1.15**	**2.54**
	CNC	0.04 (2.41)	3	0.46 (3.39)	16	1.13 (4.60)	2.54	0.96	6.54
	pCRM	0.14 (8.24)	7	1.05 (7.66)	59	4.09 (16.64)	1.71	0.96	2.76
TES [-2.5kb,0kb]	CpG	0.02 (2.24)	1	0.14 (1.77)	1	0.05 (0.35)	0.03	∼0.00	0.46
	CNC	0.01 (0.63)	1	0.12 (1.52)	3	0.20 (1.42)	13.35	∼0.00	113.00
	pCRM	0.05 (4.24)	2	0.24 (2.93)	0	0.00 (0.00)	∼0.00	∼0.00	1.10
	miRNA	0.14 (12.55)	11	1.58 (19.55)	63	4.40 (31.42)	1.57	0.94	2.44

Table S 3: **Table of descriptive statistics for each of the 5 functional annotations for the 11,446 genes of our data set.** The *cis*-candidate region was restricted to 100kb from either side of the gene. The hierarchical model was run with the TSS+TES model including the gene structure annotation. The two last sections of the table correspond to two independent analyses for which we restricted the original annotation sets to the two hotspot regions: 2.5kb from either side of the TSS and only the 2.5kb upstream the TES. The fraction in brackets are obtained by computing the corresponding denominator (e.g. number of SNP or number of eQTNs) within the subregion (otherwise the denominator refer to the entire *cis*-candidate region, i.e 100kb from either side of the gene plus the transcribed region).
