# Supplementary material for: High-Resolution Mapping of Expression-QTLs Yields Insight into Human Gene Regulation
Source: PLoS Genet. 2008 Oct 10;4(10):e1000214. doi: 10.1371/journal.pgen.1000214 (PMC2556086; doi:10.1371/journal.pgen.1000214)
Supplement: Text S1 — Supplementary methods. (0.15 MB PDF) [file pgen.1000214.s023.pdf]

High-resolution mapping of expression-QTLs yields insight into human gene regulation.

Supplementary Methods

Jean-Baptiste Veyrieras[1],
Sridhar Kudaravalli[1],
Su Yeon Kim[2],
Emmanouil T. Dermitzakis[3],
Yoav Gilad[1],
Matthew Stephens[1,2],
Jonathan K. Pritchard[1,4]

[1] Department of Human Genetics and
[2] Department of Statistics,
The University of Chicago

[3] Wellcome Trust Sanger Institute,
Wellcome Trust Genome Campus, Hinxton
[4] Howard Hughes Medical Institute.
Correspondence to jb.veyrieras@gmail.com, gilad@uchicago.edu,
mstephens@uchicago.edu, pritch@uchicago.edu.

Functional annotation of SNPs.

SNPs were annotated according to their locations within genes (see the main Methods), as well as additional annotation categories, as follow:

- CpG. The SNP lies within a CpG island according to the UCSC browser (`http://genome.ucsc.edu/`) (1; 2).
- CNC. The SNP lies within a conserved non-coding sequence (CNC) as defined using the "Most Conserved Vertebrate" track (28-way comparison) in the UCSC browser, and has no overlap with any exon (1; 2).
- CRM. The SNP falls into a predicted *cis*-regulatory module (CRM) according to the analysis of (3). The genomic locations of CRMs were obtained from `http://genomequebec.mcgill.ca/PReMod`.
- miRNA. The SNP lies within a predicted microRNA binding site, according to `http://microrna.sanger.ac.uk`, version 5 (4).
- CTCF. The SNP lies upstream of the TSS, and there is at least one predicted binding site of the CTCF insulator protein between the SNP and the TSS. To determine the genomic locations of predicted CTCF binding sites we downloaded data from `http://www.broad.mit.edu/personal/xhx/projects/CNEMOTIF/` and used the genomic locations of the motifs LM2, LM7, and LM23 (5).

These annotations were obtained using the SNP genome coordinates from the SNP physical map provided by HapMap Phase II release #21 (NCBI build 35 (hg17)). When required, the genome coordinates of the annotation tracks were converted from NCBI build 36 (hg18) to build 35 (hg17) using the Batch Coordinate Conversion tool available at UCSC web browser (6).

P-value method

Correction for spurious signals. If there is an ungenotyped SNP inside the probe of the target gene k, this SNP can affect gene expression measurements, leading to spurious variability in measured expression levels. In that case, any SNP in strong LD with the spurious SNP could produce a signal that we might interpret as an eQTL.

To correct for the effect of such spurious signals in plotting Figure 2 of the main text, we developed a correction factor based on the 634 genes for there is a genotyped HapMap SNP inside the probe. To obtain this, we masked the 634 known SNPs inside probes and computed the distribution of the most significant SNPs in LD ($r^2 > 0.5$) with the SNP inside the probe as a function of absolute distance from the probe midpoint. Of these 634

genes, only 55 were found to have a most significant SNP with p$< 7 \times 10^{-6}$ in LD with the corresponding SNP inside the probe.

Since HapMap Phase II includes ∼1/3 of the common SNPs in the three combined populations (7), we estimate that there are about 110 additional genes with significant signals that are in fact spurious. We then assume that the distribution of distances between the probes and the most significant SNPs is the same for these 110 spurious SNPs as for the 55 known spurious signals. We then used the data to predict the numbers of most-significant spurious eQTLs as a function of location relative to the probe. (We divided the 100kb region around the probe midpoint into ten 1kb bins anchored at the probe midpoint followed by nine additional 10kb bins at greater distances.) For a given distance from the probe midpoint, we can then compute the expected fraction of spurious signals by using the ratio between the expected number of spurious most significant SNPs and the observed number of most significant SNPs. (See Section 5 of the Supplement.) Finally, for the subsequent analyses, we weight each most-significant SNP by one minus the expected fraction of spurious signal according to the bin location of the most significant SNP with respect to the probe midpoint. In practice, we find that this adjustment has relatively little impact on the overall profile of observed signals.

Hierarchical model

Notation. The data consist of SNP genotypes and gene expression measurements for n individuals at each of K genes. Let y_{ik} denote the normalized gene expression data for individual i (i in $1, \ldots, n$) at gene k (k in $1, \ldots, K$). Y_k will denote the vector of gene expression values ($y_{\cdot k}$) across the n individuals at gene k.

Next, let M_k be the number of genotyped SNPs in the *cis*-candidate region of gene k. We denote the entire matrix of genotype data for these M_k SNPs with the vector G_k, and individual genotypes as g_{ijk} for individual i at SNP j of gene k. Genotypes are coded as having 0, 1, or 2 copies of the minor allele.

Bayesian regression model. As mentioned in the main paper, our hierarchical model applies the Bayesian regression framework of Servin and Stephens (8). The effect of individual i's genotype at SNP j (g_{ijk}) on their gene expression level (y_{ik}) is assumed to follow a linear model:

$$y_{ik} = \mu + a_{jk} g_{ijk} + d_{jk} I(g_{ijk} = 1) + \epsilon_{ijk} \tag{1}$$

where μ is the mean expression level at that gene for individuals with $g = 0$, and where a_{jk} and d_{jk} are the additive and dominance effects of the minor allele at SNP j. The residual, ϵ_{ijk}, is assumed to be $\mathrm{N}(0, 1/\tau)$ and

independent for each y_{ik}, where $1/\tau$ is the variance of expression levels within each genotype class. The indicator function $I(g_{ijk} = 1)$ is defined as 1 if the genotype is heterozygous ($g_{ijk} = 1$) and 0 otherwise.

Let P_k^0 denote the probability of the expression data Y_k under the null hypothesis that there are no *cis*-eQTNs in gene k (i.e., $a_{jk} = d_{jk} = 0$ for all j). Similarly, let P_{jk}^1 denote the probability of the expression data Y_k assuming that SNP j is the eQTN. In this case, the effect sizes a_{jk} and d_{jk} are modeled as being drawn from mixtures of normal distributions centered on 0 (see below for details). The Bayes factor for SNP j in gene k is defined as

$$\mathrm{BF}_{jk} = P_{jk}^1 / P_k^0, \tag{2}$$

and measures the relative support for the hypothesis that SNP j is an eQTN for gene k, versus the null hypothesis..

Prior on eQTN effect sizes. Comparing to the frequentist approach (as the p-value based model), the Bayesian regression framework allows us to investigate several kinds of regression models at the same time. We considered three distinct models for the dominance of loci, and allowed the data to estimate maximum likelihood frequencies of each type of model:

- purely additive: $a_{jk} \neq 0$ and $d_{jk} = 0$.
- additive with moderate dominance: $a_{jk} \neq 0$ and $d_{jk} \neq 0$.
- full dominance: if the derived allele is dominant then we can substitute g_{ijk} in Eq. (1) by $g_{ijk}^1 = 0, 2$ with $g_{ijk}^1 = 2$ if individual i is homozygous 11 or heterozygous. Conversely, if the derived allele is recessive we can subtitute g_{ijk} in Eq. (1) by $g_{ijk}^0 = 0, 2$ with $g_{ijk}^0 = 0$ if individual i is homozygous 11 or heterozygous. Note that by doing this we implicitly assume that $d_{jk} = 0$.

Then, as suggested by (8), we assumed that the effect sizes a_{jk} and d_{jk} are drawn from mixtures of normal distributions centered on 0 with variance σ_a^2/τ and σ_d^2/τ, respectively. Specifically, we assume a mixture of 6 normal distributions:

$$p(a_{jk}, d_{jk}|W) = \sum_{r=1}^{6} w^{(r)} N(a_{jk}; 0, \sigma_a^{(r)}/\sqrt{\tau}) N(d_{jk}; 0, \sigma_d^{(r)}/\sqrt{\tau})$$

if additive with moderate dominance

$$p(a_{jk}, d_{jk} = 0|W) = \sum_{r=1}^{6} w^{(r)} N(a_{jk}; 0, \sigma_a^{(r)}/\sqrt{\tau})$$

if purely additive or full dominance

where $N(\cdot; 0, \sigma)$ denotes the density of the normal distribution with mean 0 and standard deviation σ, $W = (w^{(1)}, \ldots, w^{(6)})$ is a vector of weights on the mixture components (where the weights are non-negative and sum to 1), and $\sigma_a^{(r)}, \sigma_d^{(r)}$ control, for each mixture component, the typical additive and dominance effects, respectively, relative to the within-genotype class standard deviation. Thus, our prior on the eQTN effect sizes can be written as follows:

$$\begin{aligned}\text{Prior}(a_{jk}, d_{jk}|\mathbf{W}, \mathbf{\Pi}) =& \Pi_{+} p(a_{jk}, d_{jk} = 0|W_{+}) + \Pi_{+d} p(a_{jk}, d_{jk}|W_{+d}) \\ &+ \Pi_{d(0)} p(a_{jk}, d_{jk} = 0|W_{d(0)}, g_{ijk} = g^0_{ijk}) \\ &+ \Pi_{d(1)} p(a_{jk}, d_{jk} = 0|W_{d(1)}, g_{ijk} = g^1_{ijk})\end{aligned}$$

where $\mathbf{W} = (W_{+}, W_{+d}, W_{d(0)}, W_{d(1)})$, $\mathbf{\Pi} = (\Pi_{+}, \Pi_{+d}, \Pi_{d(0)}, \Pi_{d(1)})$ and

- Π_{+} is the prior probability that the effect of the eQTN is purely additive,
- Π_{+d} is the prior probability that the effect of the eQTN is additive with moderate dominance,
- $\Pi_{d(0)}$ is the prior probability that the effect of the eQTN is fully dominant with allele 1 being the recessive allele,
- $\Pi_{d(1)}$ is the prior probability that the effect of the eQTN is fully dominant with allele 0 being the recessive allele,

and $\sum_{z=\{+,d+,d(0),d(1)\}} \Pi_z = 1$.

Using our hierarchical model we estimated all the weights by maximum likelihood by fixing $(\sigma_a^{(1)}, \ldots, \sigma_a^{(6)}) = (0.05,\ 0.1,\ 0.2,\ 0.4,\ 0.8, 1.6)$, and $\sigma_d^{(r)} = \sigma_a^{(r)}/4$ (this prior on d allows for moderate departures from additivity).

Bayes Factor computation. With this mixture prior, the Bayes factors (Equation 2) can be computed analytically. Specifically

$$\text{BF}_{jk} = \Pi_{+} \sum_{r=1}^{6} w^r_{+} \text{BF}^{+}_{jk}(\sigma_a^{(r)}) \tag{3}$$

$$+ \Pi_{+d} \sum_{r=1}^{6} w^r_{+d} \text{BF}^{+d}_{jk}(\sigma_a^{(r)}, \sigma_d^{(r)}) \tag{4}$$

$$+ \Pi_{d(0)} \sum_{r=1}^{6} w^r_{d(1)} \text{BF}^{d(0)}_{jk}(\sigma_a^{(r)}) \tag{5}$$

$$+ \Pi_{d(1)} \sum_{r=1}^{6} w^r_{d(0)} \text{BF}^{d(1)}_{jk}(\sigma_a^{(r)}) \tag{6}$$

where $\text{BF}^{+}_{jk}(\sigma_a)$ is the Bayes Factor evaluated at a particular value of σ_a under the purely additive regression model, $\text{BF}^{+d}_{jk}(\sigma_a, \sigma_d)$ is the Bayes Factor evaluated at a particular value of (σ_a, σ_d) under the additive with moderate dominance regression model, and $\text{BF}^{d(z)}_{jk}(\sigma^{(r)}_a)$, with $z = 0, 1$, is the Bayes Factor evaluated at a particular value of σ_a under the recessive regression model. For each model, we can derive an analytic expression for the Bayes Factor from Protocol S1, equation 13 of (8).

Modeling the gene probability. Our hierarchical model assumes that there are two mutually exclusive categories of genes. With probability Π^{snp}_k gene k has a SNP inside the probe and with probability $1 - \Pi^{\text{snp}}_k$ gene k has no SNP inside the probe. We define Π^{snp}_k as follows:

$$\Pi^{\text{snp}}_k = \begin{cases} 1 & \text{if gene } k \text{ has a genotyped SNP inside the probe} \\ \frac{N_s(1/\alpha-1)}{N-N_s} & \text{otherwise.} \end{cases}$$

where N_s is the number of genes for which we observed a SNP inside the probe (N_s =634 in our dataset), N the total number of genes (here $N = 11446 + 634 = 12080$) and α the fraction of actual SNPs which have been genotyped ($\alpha \approx 1/3$ for the combined populations of HapMap Phase II), so that $\frac{N_s(1/\alpha-1)}{N-N_s}$ gives us an estimate of the fraction of genes for which there could be a SNP inside the probe in the set of genes without an observed SNP inside the probe (i.e $N - N_s = 11446$).

Then the probability of the expression data for gene k can be written as

$$\Pr(Y_k) = \Pi^{\text{snp}}_k(\Pi_s P^{\text{S}}_k + (1 - \Pi_s)P^{\text{R}}_k) + (1 - \Pi^{\text{snp}}_k)P^{\text{R}}_k \quad (7)$$

where P^S_k denotes the probability of the expression data Y_k given that there is a SNP inside the probe leading to exactly one spurious eQTN, P^{R}_k is the probability of the expression data Y_k given that the probe signal is not altered by a SNP inside the probe, and Π_s is the probability that when a gene has a SNP inside the probe this creates a spurious eQTN.

When we run the hierarchical model, we actually include the 634 genes with a known SNP in the probe. For these genes, we set $\Pi^{\text{snp}}_k = 1$. These genes then provide training data from which to estimate Π_s and the distribution of locations of spurious SNPs, relative to the probe location (parameterized by β and γ, defined below).

Modeling the probability to be a genuine eQTN. Given that the probe signal is not altered by a SNP inside the probe, we consider two mutually exclusive categories of genes: with probability Π_0 there is no genuine eQTN in the *cis*-candidate region and with probability $\Pi_1 = 1 - \Pi_0$ there is a genuine eQTN:

$$P^{\text{R}}_k = \Pi_0 P^0_k + \Pi_1 P^1_k \quad (8)$$

where P_k^0 denotes the probability of the expression data Y_k given that there is no genuine eQTN in gene k and P_k^1 denotes the probability of the expression data given that there is exactly one genuine eQTN.

Given that there is a single genuine eQTN in gene k, the probability of the observed expression data, P_k^1, can be written as

$$P_k^1 = \sum_{j=1}^{M_k} \pi_{jk} P_{jk}^1 \tag{9}$$

where P_{jk}^1 is the probability of the expression data given that SNP j is an eQTN, and π_{jk} is the (prior) probability that SNP j is an eQTN, given that exactly one SNP in gene k is an eQTN.

A key feature of the hierarchical model is that the probability that SNP j is an eQTN, π_{jk}, is allowed to depend on the physical location of SNP j relative to one or more "anchor" points, and other relevant annotations. Suppose that we consider L different kinds of annotation, and let the indicator δ_{jkl} equal 1 if SNP j at gene k has the lth annotation, and equal 0 otherwise. Then define

$$x_{jk} = \sum_{l=1}^{L} \lambda_l \delta_{jkl}, \tag{10}$$

where $\Lambda = (\lambda_1, \ldots, \lambda_L)$ is a vector of annotation effect parameters. We use a logistic model to relate π_{jk} to these annotation indicators, namely,

$$\pi_{jk} = \frac{\exp(x_{jk})}{\sum_{j'=1}^{M_k} \exp(x_{j'k})}. \tag{11}$$

Modeling the probability to be a spurious eQTN. Similarly to the modeling of the probability to be a genuine eQTN, we defined P_k^{S}, the probability of the expression data given that there is an untyped SNP in the probe that creates a spurious eQTN, as follows:

$$P_k^{\mathrm{S}} = \sum_j \pi_{jk}^s P_{jk}^s \tag{12}$$

where

$$\pi_{jk}^s = \frac{\exp(\phi(d_{jk}))}{\sum_{j'=1}^{M_k} \exp(\phi(d_{j'k})))}, \tag{13}$$

d_{jk} is the distance between SNP j in gene k and the midpoint of the probe and $\phi(.)$ an appropriate function which should reflect the shape of LD decay with distance. Here, we assumed $\phi(x) = \beta \exp(-\gamma|x|)$ to be a reasonable candidate, with (β, γ) being considered as model parameters.

Likelihood for the hierarchical model. Substituting the above expressions for P_k^{S} and P_k^{R}, into (7) and after some rearrangements, the likelihood for the hierarchical model is

$$\begin{aligned}
\Pr(Y_k|\Theta) &= \Pi_0^* P_k^0 + \Pi_{ks} \sum_{j=1}^{M_k} \pi_{jk}^s P_{jk}^s + \Pi_1^* \sum_{j=1}^{M_k} \pi_{jk} P_{jk}^1 \qquad (14) \\
&= P_k^0 \left(\Pi_0^* + \Pi_{ks} \sum_{j=1}^{M_k} \pi_{jk}^s \mathrm{BF}_{jk}^s + \Pi_1^* \sum_{j=1}^{M_k} \pi_{jk} \mathrm{BF}_{jk}^1 \right) \qquad (15)
\end{aligned}$$

where:

- $\Pi_{ks} = \Pi_k^{\mathrm{snp}} \Pi_s$ is the prior probability that there is a spurious eQTN given that there is a SNP inside the probe,
- $\Pi_0^* = (1 - \Pi_{ks})\Pi_0$ is the prior probability that there is no genuine eQTN given that there is no SNP inside the probe leading to a spurious eQTN,
- $\Pi_1^* = (1-\Pi_{ks})\Pi_1$ is the prior probability that there is a genuine eQTN given that there is no SNP inside the probe leading to a spurious eQTN,
- Θ denotes the model parameters
- BF_{jk}^s is the BF from the Bayesian regression given that the SNP is a spurious eQTN,
- BF_{jk} is the BF from the Bayesian regression given that the SNP is a genuine eQTN.

To be explicit, the model parameters Θ include the annotation parameters Λ, the parameters of the spurious term (β, γ), the mixture weights W_s for the BF under the spurious model (and we assume that spurious eQTNs have only purely additive effects, an assumption that has empirical support from the 634 genes with a HapMap SNP inside the probe), the mixture weights $(\mathbf{W}, \mathbf{\Pi})$ for the BF under the genuine eQTN model, and the proportions Π_0 and Π_s. The likelihood of the entire data set is the product of (15) across all K genes. We fit the hierarchical model by maximizing the log-likelihood

$$\mathrm{L}(Y|\Theta) = \sum_{k=1}^{K} \log(P_k^0) \; + \sum_{k=1}^{K} \log \left(\Pi_0^* + \Pi_{ks} \sum_{j=1}^{M_k} \pi_{jk}^s \mathrm{BF}_{jk}^s + \Pi_1^* \sum_{j=1}^{M_k} \pi_{jk} \mathrm{BF}_{jk}^1 \right) \qquad (16)$$

with respect to the model parameters Θ. (Note that the first term, involving P_k^0 does not depend on Θ, and so need not be evaluated.)

Likelihood maximization. To maximize (16) we used an iterative strategy based on a point-by-point golden maximization strategy (9). To speed convergence of the maximization process, we initialized the parameters using naive estimates of the λs based on the logarithm of the odds ratio computed assuming $\Pi_0 = \Pi_s = 0$ but only on the subset of genes for which there is no HapMap SNP inside the probe. We then force the algorithm to start with $\Pi_s = 0.99$ (i.e that we initially assume that 99% of the genes with a SNP inside the probe have a spurious eQTN). The parameters of the spurious model (β, γ) are also the first parameters to be estimated at each iteration of our maximization algorithm.

Posterior probabilities. Once the likelihood has been maximized, we can compute the posterior probability of a given SNP j to be either an eQTN for gene k:

$$\Pr(\text{SNP } j \text{ is an eQTN for gene } k|Y_k, \hat{\Theta}) = \frac{\hat{\Pi}_1^* \hat{\pi}_{jk} BF_{jk}}{\Pr(Y_k|G_k, \hat{\Theta})} \quad (17)$$

or a spurious eQTN due to an unobserved SNP inside the target probe:

$$\Pr(\text{SNP } j \text{ is spurious eQTN for gene } k|Y_k, \hat{\Theta}) = \frac{\hat{\Pi}_{ks} \hat{\pi}^s_{jk} BF_{jk}}{\Pr(Y_k|G_k, \hat{\Theta})}. \quad (18)$$

Confidence interval of the parameters. After computing maximum likelihood estimates of the model parameters, we constructed confidence intervals for each parameter in turn from the log-likelihood curve by including all values of the parameter θ_i for which $\log(\mathrm{L}(Y|\hat{\Theta}_{-i}; \theta_i))$ is within 2 units of the maximum, fixing $\hat{\Theta}_{-i} = \hat{\Theta} - \{\hat{\theta}_i\}$. Under standard asymptotic theory, confidence intervals constructed in this way would include the true value of $\theta_i \approx 95\%$ of the time (this follows from the asymptotic χ^2 distribution of the log-likelihood-ratio statistic).

Testing for symmetry of signals at the TSS and TES. To assess whether the signal peaks at the TSS and TES were biased either upstream or downstream, we used the hierarchical model with ten 1kb bins on either side of the anchor point (TSS and TES). We computed likelihoods under a model where the 1kb bins immediately upstream and downstream of the TSS (respectively TES) were (1) forced to use the same λ and (2) allowed to use different values of λ. Under the null hypothesis (symmetry around the TSS or TES), twice the difference in log likelihood between model (2) and model (1) should follow a $\chi^2(1)$ distribution.

Partitioning the *cis*-candidate region around an anchor point. Recall that in our analyses we considered only genes lower than 500kb and a

cis-candidate region of 500kb from either side of the target gene plus the gene itself. So, for each anchor point the partition has to cover the entire *cis*-candidate region (i.e 1.5Mb). Depending on the anchor point we proceeded as follows:

- TSS: the 500kb region upstream the TSS is divided into four bins of 100kb anchored at -500kb from the TSS, followed by nine bins of 10kb and ten bins of 1kb. The 1Mb region downstream the TSS is then divided in ten bins of 1kb anchored at the TSS, followed by nine bins of 10kb and nine bins of 100kb.
- TES: the 1Mb region upstream the TES is divided into nine bins of 100kb anchored at -1Mb from the TES, followed by nine bins of 10kb and ten bins of 1kb. The 500kb region downstream the TES is then divided in ten bins of 1kb anchored at the TES, followed by nine bins of 10kb and four bins of 100kb.
- Other anchors (CDSS, CDSE, CDSMID, TXMID, PRBMID): since for these anchors the size of the upstream or downstream region varies depending on the position of the anchor point within the transcribed region, we used a quasi-symmetrical partition around the anchor depending on the bias of the anchor: if the anchor is 5' biased (CDSS and CDSMID), we considered the upstream region to span 800kb otherwise 700kb. Then we used the same strategy as for the TSS, TES: 100kb bins after a distance of 100kb from the anchor and nine 10kb bins followed by ten 1kb bins for the 100kb region flanking the anchor (on each side). For the TXMID (transcipt midpoint) we used a symmetric partition with 750kb on either side of the anchor and two 150kb bins at each end.

Note that whatever the anchor point, these partition schemes yield exactly 51 bins leading to 51 distinct λ's in the hierarchical model. So, for example, the TSS-only model which includes only the TSS anchor point has 51 bin parameters meanwhile the TSS+TES model which consider both the TSS and the TES anchor points has $51 \times 2 = 102$ bin parameters.

Simulations

To investigate the properties of our methods, we developed the following simulation scheme. We used the real HapMap data, and simulated the expression data, conditional on the genotypes, as follows:

- a gene has a single eQTN with probability Π_1,
- the location of this single eQTN depends only on its distance from the TSS, whatever the gene size,

- the effect on gene expression of the simulated eQTN is purely additive,
- the distribution of the proportion of variance explained by the simulated eQTNs follows the observed distribution,
- only a fraction α of these eQTNs are genotyped in the simulated data set.

To simulate such a dataset we looped on the gene transcripts of the original dataset and proceeded as follows:

1. draw with probability $\Pi_1 = 0.20$ if gene has an eQTN. Otherwise discard the gene.
2. using a Laplacian distribution centered around the TSS draw the eQTN location. The Laplacian distribution mimics a symmetrical exponential decay of the probability to be an eQTN around the TSS. In practice, we used a discretized version of this distribution using 1kb bins spanning the entire region (i.e the gene region plus 500kb from either side of the gene). Then, we picked with the corresponding probability the 1kb bin eligible to contain the eQTN. If the bin didn't contain any HapMap SNPs, we repeated the previous step until picking a bin with at least one HapMap SNP inside. We then randomly chose a HapMap SNP inside the bin to be the eQTN.
3. draw π the proportion of variance explained by the eQTN from the observed distribution.
4. compute the eQTN effect size, namely a, by using the following approximation: $a \approx \sqrt{\frac{\pi}{(1-\pi)f(1-f)}}$, where f is the derived allele frequency.
5. simulate the gene expression levels, $\{y_i\}$, for the 210 HapMap individuals as follows: $y_i \sim \mathcal{N}(a * g_i, \sigma_e)$ where $g_i = \{0, 1, 2\}$ is the eQTN genotype of individual i and $\sigma_e = 1 - \pi$ is the environmental variance.
6. with probability $1 - \alpha$ mask the HapMap SNP corresponding to the eQTN. If the SNP is masked it will be ignored in the subsequent analyses. The results plotted use either $\alpha = 1$ (complete genotype data) or $\alpha = 0.3$, which is an estimate of the fraction of true eQTNs in the real data since HapMap Phase II is estimated to contain around 30% of the total number of common SNPs in the three combined populations.

Spurious signal

Figure S19 illustrates the idea of the correction used in Figure 2 of the main paper in order to control for any confouding effect due to spurious

signal from HapMap SNP in LD with an unobserved SNP inside the probe. The assumptions underlying this correction are: i) if there is an ungenotyped SNP inside the probe, any HapMap SNP in moderate or strong LD with that SNP can generate a spurious eQTL, ii) the probability to observe a spurious eQTL then mainly depend on the extend of LD from either side of the probe midpoint, iii) the 634 genes for which we observe a HapMap SNP inside the probe constitute a good subset of genes to learn about the probability that a SNP inside the probe affects the gene expression measurement and also about the probability to find a spurious eQTL as a function of distance from the probe midpoint.

In Figure S19 we plotted signal as a function of absolute distance from the probe midpoint based on the 11,446 genes without a HapMap SNP inside the probe and the 634 genes with at least one HapMap SNP inside the probe. In each panel the black part of the bars correspond to the number of most significant SNPs ($p < 7 \times 10^{-6}$) in moderate LD ($r^2 > 0.5$) with the SNP inside the probe for the corresponding 634 genes (the SNP inside the probe being masked). The red and green parts of the bar correspond to the number of most significant SNPs observed in the remaining 11,446 genes without a HapMap SNP inside the probe. Since HapMap contains only $\sim 1/3$ of common SNPs, the red fraction of each bar is roughly equal to twice the black fraction, and thus corresponds to the fraction of signal expected to be spurious due to an unoberved SNP inside the probe (among the 11,446 genes). Thus the green part represents the fraction of eQTLs that we expect to be genuine. So that, in a given bin, taking the ratio between the red part, namely r, and the red plus the green parts, namely $r + g$, gives us the expected fraction of spurious eQTLs in that bin ($e \approx r/(r+g)$). Consequently, the contribution to further analyses of each eQTLs falling in that bin has to be weighted by one minus this fraction (i.e $1 - e$ which can be interpreted as the probability that the SNP is a genuine eQTL).

Note that assuming that all the genuine eQTLs inside the 1kb bin around the probe midpoint are in fact spurious is pretty unlikely as this will mean that only 10% of the common SNPs are genotyped in that bin. But we know that ~95% of the probes are located into the last exon of the target genes, a region which is likely to exhibit a positive ascertainment bias as suggested by its high SNP density (see Table S2).

References

[1] Kent W, Sugnet C, Furey T, Roskin K, Pringle T, et al. (2002) The human genome browser at UCSC. Genome Res 12:996–1006.

[2] Kuhn R, Karolchik D, Zweig A, Trumbower H, Thomas D, et al. (2007) The UCSC genome browser database: update 2007. Nucleic Acids Res 35:D668–73.

[3] Blanchette M, Bataille A, Chen X, Poitras C, Laganiere J, et al. (2006) Genome-wide computational prediction of transcriptional regulatory modules reveals new insights into human gene expression. Genome Res 16:656–668.

[4] Griffiths-Jones S, Grocock R, van Dongen S, Bateman A, Enright A (2006) miRBase: microRNA sequences, targets and gene nomenclature. Nucleic Acids Res 34:D140–144.

[5] Xie X, Mikkelsen T, Gnirke A, Lindblad-Toh K, Kellis M, et al. (2007) Systematic discovery of regulatory motifs in conserved regions of the human genome, including thousands of CTCF insulator sites. Proc Natl Acad Sci U S A 104:7145–50.

[6] Hinrichs A, Karolchik D, Baertsch R, Barber G, Bejerano G, et al. (2006) The UCSC Genome Browser Database: update 2006. Nucleic Acids Res 34:D590–8.

[7] International HapMap Consortium (2007) A second generation human haplotype map of over 3.1 million SNPs. Nature 449:851–861.

[8] Servin B, Stephens M (2007) Imputation-based analysis of association studies: candidate regions and quantitative traits. PLoS Genet 3:e114.

[9] Brent R (1973) Algorithms for Minimization without Derivatives. New Jersey: Prentice Hall.
